# TaPP2C‐a6 interacts with TaDOG1Ls and regulates seed dormancy and germination in wheat

**DOI:** 10.1111/pbi.70144

**Published:** 2025-05-26

**Authors:** Qian Zhang, Xiaofen Yu, Ruibin Wang, Ya'nan Wu, Fu Shi, Yufan Zhang, Hongyan Zhao, Huazhen Xu, Jiao Pan, Yuesheng Wang, Min Tu, Junli Chang, Zhanwang Zhu, Guangyuan He, Mingjie Chen, Ling Chen, Guangxiao Yang, Yin Li

**Affiliations:** ^1^ The Genetic Engineering International Cooperation Base of Chinese Ministry of Science and Technology, Key Laboratory of Molecular Biophysics of Chinese Ministry of Education College of Life Science and Technology, Huazhong University of Science and Technology Wuhan China; ^2^ Key Laboratory of Plant Germplasm Enhancement and Specialty Agriculture, Wuhan Botanical Garden, Innovative Academy of Seed Design Chinese Academy of Sciences Wuhan China; ^3^ Hubei Technical Engineering Research Center for Chemical Utilization and Engineering Development of Agricultural and Byproduct Resources, School of Chemical and Environmental Engineering Wuhan Polytechnic University Wuhan China; ^4^ Institute of Food Crops Hubei Academy of Agricultural Sciences Wuhan China; ^5^ Hubei Key Laboratory of Food Crop Germplasm and Genetic Improvement Wuhan China; ^6^ Key Laboratory of Crop Molecular Breeding Ministry of Agriculture and Rural Affairs Wuhan China

**Keywords:** wheat, seed dormancy, pre‐harvest sprouting, ABA signalling, clade‐A *PP2Cs*, *DOG1*

## Abstract

Modern wheat cultivation requires seed to germinate rapidly and uniformly with weak dormancy. However, such varieties tend to undergo pre‐harvest sprouting (PHS) if the harvest overlaps with the rainy season, causing substantial yield losses. Knowledge regarding the mechanisms of seed dormancy in wheat (*Triticum aestivum* L.) is limited, with only a few causal genes of the many PHS quantitative trait loci (QTLs) characterized. Here, we emphasize the involvement of ABA signalling core components in regulating seed dormancy and germination in wheat. *TaPP2C‐a6* was identified as the likely causal gene of wheat *PHS‐QTLs QPhs.wsu‐1A/1B* and *QPhs1D.1_nwafu* loci. Both *TaPP2C‐a6* and *TaPP2C‐a7* were highly expressed at embryonic developmental stages and germinating seeds, whereas *TaPP2C‐a6* was up‐regulated during embryo maturation and seed germination. TaPP2C‐a6 and TaPP2C‐a7 were clade‐A PP2Cs that interacted with TaPYLs and class III TaSnRK2s; however, TaPP2C‐a6 showed stronger interactions with TaDOG1L members than those of TaPP2C‐a7. *TaPP2C‐a6* overexpression in transgenic *Arabidopsis thaliana* caused a more severe reduction in ABA sensitivity than *TaPP2C‐a7* overexpression. Overexpression of *TaPP2C‐a6* in transgenic *A. thaliana* and wheat increased PHS levels, whereas *TaPP2C‐a7* transgenic *A. thaliana* did not affect PHS levels, confirming that TaPP2C‐a6 is a novel regulator of wheat seed dormancy and germination. In summary, we demonstrated that leveraging the knowledge of seed dormancy and germination from model species could rapidly identify the causal genes of PHS‐QTLs in wheat. Significantly, we showed that the TaPP2C‐TaDOG1L interactions, particularly the interaction strength, could be a new aspect in the regulation of seed dormancy and germination.

## Introduction

Seed dormancy and germination are two related physiological processes crucial for the complete life cycles of seed plants and their geographical distributions (Cantoro *et al*., 2013). During seed maturation, dormancy is established to prevent germination under unfavourable conditions, while during germination, the seed transits from a dormant to an active physiological state (Carrillo‐Barral *et al*., 2020; Graeber *et al*., 2012; Wang *et al*., 2014b). The establishment and release of seed dormancy affect rapid and uniform seed germination and are related to subsequent vegetative growth and crop yield. Therefore, seed dormancy and germination traits are selected during crop domestication and improvement. High levels of seed dormancy could delay seed germination and possibly result in a shortened growth period under favourable conditions, which may affect appropriate harvest during cultivation, thereby influencing crop yield and quality (Shu *et al*., 2016). In contrast, low levels of seed dormancy often lead to pre‐harvest sprouting (PHS), causing substantial losses in crop production (Fang and Chu, 2008; Sato and Kohler, 2022; Tai *et al*., 2021). PHS refers to the phenomenon in which mature seeds sprout from the mother plant before harvesting during continuous rainy and humid weather (Miao *et al*., 2019; Sohn *et al*., 2021). Therefore, it is important to accurately regulate dormancy levels to prevent PHS and ensure uniform and ready germination to secure cereal crop yields.

Seed dormancy and germination are precisely regulated by multiple environmental cues, such as water (Liu *et al*., 2019), light (Hoang *et al*., 2014; Seo *et al*., 2009; Yang *et al*., 2020), temperature (Rodríguez *et al*., 2015; Springthorpe and Penfield, 2015), and endogenous phytohormones, particularly abscisic acid (ABA) and gibberellic acid (GA) (Finkelstein *et al*., 2008; Holdsworth *et al*., 2008; Rodríguez‐Gacio Mdel *et al*., 2009). ABA and GA are the two major phytohormones acting antagonistically in regulating seed dormancy and germination. The ABA content gradually increases in seeds during maturation to promote the establishment of seed dormancy. During seed germination, the GA content gradually increases to activate a series of physiological processes required for germination (Lang *et al*., 2021; Luo *et al*., 2021; Penfield, 2017; Shu *et al*., 2016; Sohn *et al*., 2021). Other phytohormones, such as auxin (AUX), cytokinins (CTK), ethylene (ETH), brassinosteroid (BR), salicylic acid (SA), jasmonic acid (JA), and strigolactone (SL), indirectly regulate seed dormancy and germination by interacting with ABA/GA metabolism and signalling pathways (Belin *et al*., 2009; Ju *et al*., 2019; Liu *et al*., 2013; Ramaih *et al*., 2003; Sun *et al*., 2020; Tai *et al*., 2021; Tong *et al*., 2014; Unterholzner *et al*., 2015). Therefore, maintaining a dynamic balance between ABA and GA and ABA/GA signalling pathways is important for the precise regulation of seed dormancy and germination in plants.

The core components of ABA signalling have been well identified in plants, including the ABA receptor (pyrabactin resistance 1 (PYR1)/PYR1‐like (PYL)/regulatory component of ABA receptor (RCAR)) (hereafter referred to as PYL), the clade‐A type‐2C protein phosphatase (PP2C), the ABA‐activating class III SNF1‐related protein kinase 2 (SnRK2) and ABA‐responsive element binding factor (AREB/ABF) (reviewed in Chen *et al*., 2020). Notably, the members from almost all ABA signalling core component families are involved in regulating seed dormancy and germination. For example, reducing the activity of clade‐A PP2Cs (e.g. ABI1 and ABI2) activates class III SnRK2s, which phosphorylate and activate the downstream transcription factors AREB/ABFs to promote seed dormancy (Merlot *et al*., 2001; Umezawa *et al*., 2009; Wang *et al*., 2020b). In Arabidopsis, class III SnRK2s positively regulate seed dormancy, and the seeds of *snrk2.2/2.3/2.6* triple mutant lose dormancy (Nakashima *et al*., 2009). In addition, wheat TaMYB10 regulates the ABA biosynthetic gene *NCED* to confer PHS resistance (Lang *et al*., 2021).

In addition to the ABA signalling core components, many other genes regulate seed germination and dormancy, which have been well reviewed (Iwasaki *et al*., 2022; Penfield, 2017; Shu *et al*., 2016; Tai *et al*., 2021). Among these genes, *Delay of Germination 1* (*DOG1*) and *DOG1‐like* (*DOG1L*) genes encode a plant‐specific family of small proteins with major functions in the regulation of seed dormancy. *DOG1* was first identified as the causal gene of a major quantitative trait locus (QTL) that positively regulates seed dormancy in *Arabidopsis thaliana* (Bentsink *et al*., 2006). However, the detailed molecular functions of DOG1 remain elusive until recently. Recent studies have shown that DOG1 interacts with AHG1/AHG3 and negatively regulates PP2C activity to control seed dormancy and germination (Nee *et al*., 2017a; Nishimura *et al*., 2018). DOG1‐AHG1/AHG3 constitutes an important regulatory hub of seed dormancy and germination, which converges ABA signalling‐mediated dormancy regulation with clade‐A PP2Cs (Nee *et al*., 2017b; Umezawa *et al*., 2010). Our group previously identified six TaDOG1L proteins in wheat (Yu *et al*., 2020). The ectopic expression of *TaDOG1L*s and *HvDOG1L*s in Arabidopsis promoted seed dormancy (Ashikawa *et al*., 2010, 2013). Overexpression of *TaDOG1L4* and *HvDOG1L1* in wheat enhanced seed dormancy, whereas the RNAi‐mediated knockdown of *TaDOG1L4* repressed seed dormancy (Ashikawa *et al*., 2014). These results suggested that the functions of the *DOG1* family in seed dormancy are conserved between Arabidopsis and cereal crops. In addition, *REDUCED DORMANCY 5* (*RDO5*) encodes a seed‐specifically expressed PP2C without phosphatase activity and acts as a pseudophosphatase to control seed dormancy by preventing dephosphorylation during seed imbibition (Xiang *et al*., 2014).

Wheat varieties have been domesticated and selected for their relatively weak seed dormancy to allow rapid and near‐simultaneous germination to optimize cultivation. However, such selection tends to have low levels of dormancy, frequently causing PHS in the rainy season, which often overlaps with the harvest season in many areas of the major wheat‐growing countries (for example, Canada, Australia and China) (Kulwal *et al*., 2012; Sydenham and Barnard, 2018; Zhou *et al*., 2017). Therefore, the development of PHS‐tolerant wheat varieties is urgently required. Currently, identifying genes conferring PHS tolerance and breeding applications in wheat relies primarily on QTL mapping and the cloning of causal genes. Some PHS‐resistance QTLs have been identified in the wheat germplasm (Tai *et al*., 2021, 2024). However, our knowledge of the mechanisms that control seed dormancy in wheat is limited. PHS‐resistant genetic resources often lack sufficient utilization during wheat breeding programs because only a few causal genes of the wheat PHS‐QTL loci were cloned with the gene functions studied, for example, *R‐1* (*TaMYB10*) (Himi *et al*., 2011; Lang *et al*., 2021), viviparous‐1 (*TaVp‐1*) (Yang *et al*., 2007), jasmonate‐ZIM domain 1 (*TaJAZ1*) (Ju *et al*., 2019) and phosphatidylinositol 4‐kinase (*TaPI4K*) (Tai *et al*., 2024). Knowledge of the molecular regulation of seed dormancy in Arabidopsis and wheat indicates that many ABA‐signalling core components are likely involved in this process.

In this study, we provided a straightforward approach to rapidly identify key genes within wheat PHS‐QTL loci. We demonstrated that *TaPP2C‐a6* is likely the causal gene of *QPhs.wsu‐1A/1B* and *QPhs1D.1_nwafu* loci. Both *TaPP2C‐a6* and *TaPP2C‐a7* are highly expressed during seed maturation and affect ABA sensitivity; however, *TaPP2C‐a6* positively regulates seed germination, and its overexpression leads to PHS in both Arabidopsis and wheat plants. Importantly, we showed that TaPP2C‐a6 interacted more strongly with TaDOG1Ls than that of TaPP2C‐a7, which may represent a new regulatory aspect of seed germination and dormancy in wheat.

## Results

### TaPP2C‐a6 is a candidate of the causal gene for the QPhs.Wsu‐1A.2 and QPhs.Wsu‐1B.2 loci

**Figure 1:**
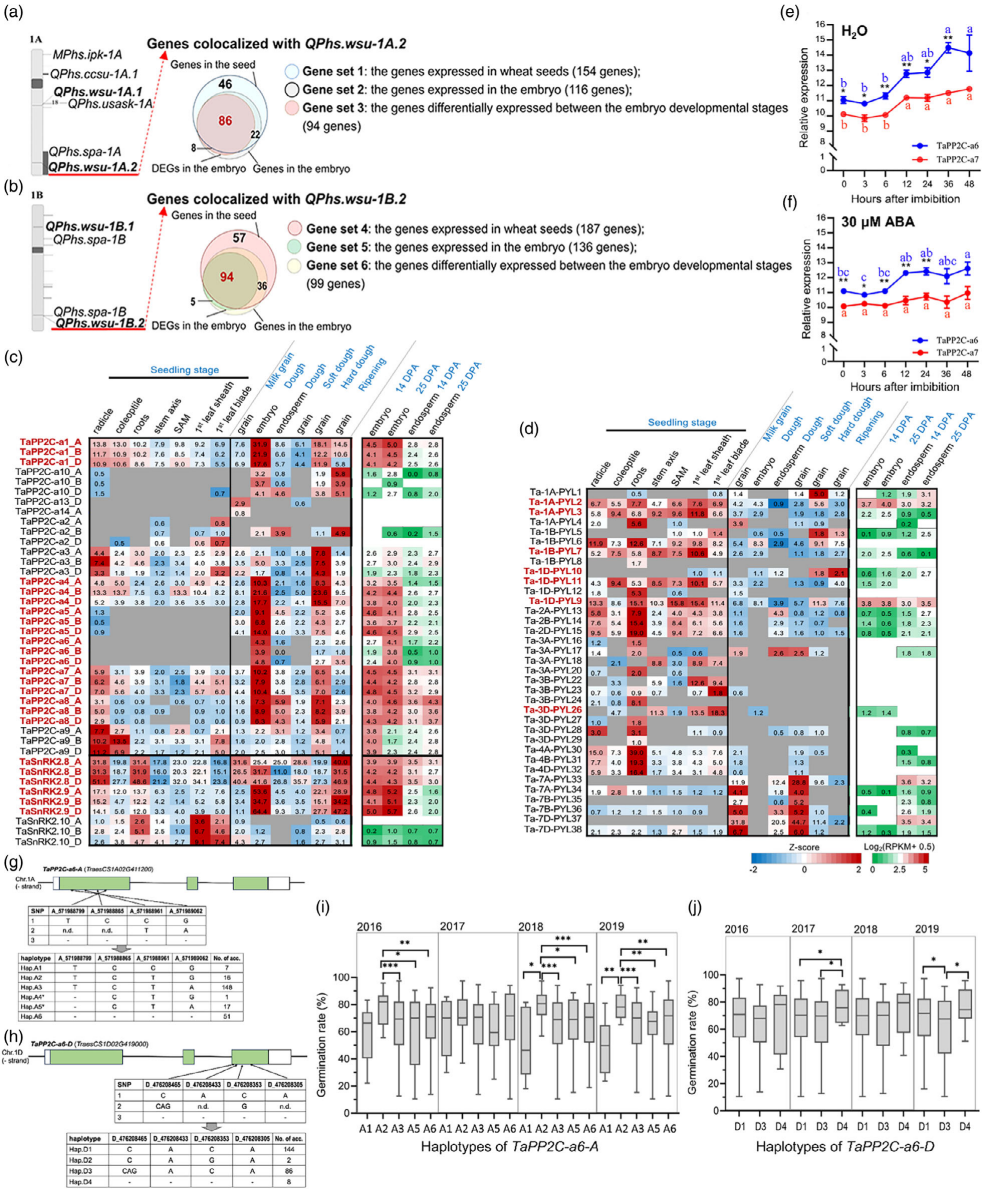
Analyses of the genes encoding ABA‐signalling core components in wheat identify *TaPP2C‐a6‐1A* and *TaPP2C‐a6‐1B* as the top candidate genes for the PHS‐QTL loci. (a, b) *TaPP2C‐a6‐1A* and *TaPP2C‐a6‐1B* were found to be the top candidates of the causal genes for the *QPhs.wsu‐1A.2* (a) and *QPhs.wsu‐1B.2* (b) locus, respectively, that were differentially expressed in embryo developmental stages. (c, d) The spatial–temporal expression profiles of the ABA‐signalling genes highlighted the gene members of *TaPYLs*, clade‐A *TaPP2Cs* and class III *TaSnRK2s* that were highly expressed during seed development and germination (gene names shown in red) (Ramírez‐González *et al*., 2018; Wei *et al*., 2019). The results are shown in heat maps: for the expression data from Ramírez‐González *et al*. (2018), grey indicates not expressed, while blue and red indicate low and high expression levels; for the expression data from Wei *et al*. (2019), white indicates not expressed, while green and red indicate low and high expression levels. The expression levels (in RPKM) are labelled. The tissues are labelled in black and stages labelled in blue. (e, f) qPCR‐based expression patterns of *TaPP2C‐a6* and *TaPP2C‐a7* during seed germination time points with (f) or without ABA (e). The expression data are presented as means ± standard error of the mean (SEM) of three biological replicates. Statistical differences of expression levels between the time points within the same gene were calculated with ANOVA by using SPSS software and indicated with letter presentation (determined by *post‐hoc* Tukey's test), while the statistical differences between *TaPP2C‐a6* and *TaPP2C‐a7* for the same time point were determined by Student's *t*‐test (* and ** standing for *P* < 0.05 and *P* < 0.01, respectively). (g, h) Exon–intron structure of *TaPP2C‐a6‐1A* (g) and *TaPP2C‐a6‐1D* (h) and sequence polymorphisms detected within the 240‐accession GWAS population. (i, j) Boxplots of PHS germination rates based on the haplotypes for *TaPP2C‐a6‐1A* (i) and *1D* (j) from 2016 to 2019. Significant differences between the haplotypes were analysed by Welch's *t*‐test (*, ** and *** standing for *P* < 0.05, *P* < 0.01 and *P* < 0.001, respectively).

Genes encoding ABA‐signalling core components have been characterized in wheat, including 38 genes encoding ABA receptors (*PYLs*), 36 genes encoding clade‐A *PP2Cs*, and 9 genes encoding class III *SnRK2s* (Lei *et al*., 2021; Mao *et al*., 2022; Mega *et al*., 2019; Yu *et al*., 2019; Zhang *et al*., 2023) (Table S1). Since many members of these ABA‐signalling gene families regulate seed dormancy and germination in model plant species, we investigated whether these genes represent a list of candidate causal genes when mining the PHS‐QTL loci of wheat. A systematic search of the wheat PHS‐QTL loci based on the wheat QTL atlas (Singh *et al*., 2021) and published literature (Li *et al*., 2021; Lin *et al*., 2017; Martinez *et al*., 2018; Tai *et al*., 2024; Zhou *et al*., 2017; Zhu *et al*., 2019) identified that *TaPP2C‐a6‐1A*, *TaPP2C‐a6‐1B*, and *TaPP2C‐a6‐1D* were co‐localized within the linked regions of the PHS loci *QPhs.wsu‐1A.2*, *QPhs.wsu‐1B.2*, and *QPhs1D.1_nwafu*, respectively (LD length = 10 Mb was used according to previous wheat population genomics studies (Hao *et al*., 2020; Pang *et al*., 2020)). The PHS loci *QPhs.wsu‐1A.2* and *QPhs.wsu‐1B.2* were identified by Martinez *et al*. (2018), while the *QPhs1D.1_nwafu* locus was identified by Tai *et al*. (2024). A series of expression analyses were performed to prioritize the candidate genes within the two QTL loci: (1) the high‐confidence gene models within the LD decay regions for each locus were retrieved; (2) the candidate causal genes should be expressed during either embryo developmental or seed‐germinating stages; (3) the candidate causal genes may be differentially expressed during embryo development. Our expression analyses identified 324 and 375 genes expressed within the *QPhs.wsu‐1A.2* and *QPhs.wsu‐1B.2* loci, respectively (Tables S2 and S3), and 86 and 94 differentially expressed genes (DEGs) during embryonic development were considered candidates for the causal genes of the QTL loci (Figure 1a,b). Functional annotations for the 86 and 94 genes highlighted that the Chinese Spring RefSeqv1.1 *TraesCS1A02G411200* and *TraesCS1B02G441400* encode TaPP2C‐a6‐1A and TaPP2C‐a6‐1B, respectively, both of which are expressed in seeds and up‐regulated during embryo stages based on RNA‐seq data (Wei *et al*., 2019) (Tables S4 and S5). In addition, the expression analysis showed that 27 *PYL*‐encoding genes, 30 *PP2C*‐encoding genes, and 9 *SnRK2*‐encoding genes were expressed, respectively, during embryonic development (Figure 1c,d). Several genes encoding the clade‐A *TaPP2Cs* and class III *TaSnRK2s* were expressed in seeds. Among them, the genes encoding TaPP2C‐a1/a4/a5/a6/a7/a8 and TaSnRK2.8/2.9 were highly expressed during seed development stages, indicating that they could play important roles in the regulation of seed dormancy (Figure 1c). In particular, *TaPP2C‐a6* genes were the only *PP2C* genes up‐regulated in the embryonic stages (Figure 1c), suggesting that they may play a role in ABA signalling in wheat embryos and the establishment of seed dormancy. Phylogenetic analysis of clade‐A TaPP2Cs showed that TaPP2C‐a6 belongs to the AHG1 subgroup (Figure S1a), of which other *AHG1 PP2C* genes in Arabidopsis regulate seed dormancy and germination. Besides, our qRT‐PCR experiments demonstrated that both *TaPP2C‐a6‐1D* and *TaPP2C‐a7‐3D* were expressed within the 48 h of seed imbibition with or without 30 μM ABA, and *TaPP2C‐a6‐1D* was up‐regulated with a higher expression level than that of *TaPP2C‐a7‐3D* (Figure 1e,f). Additional qRT‐PCR analysis confirmed that *TaPP2C‐a6‐1D* had the highest expression levels in the embryo and endosperm, with very high levels of expression detected at 25 days post‐anthesis (dpa) (Figure S1b,c). Collectively, our results indicate the role of *TaPP2C‐a6* in the ABA signalling during seed maturation and germination, highlighting it as a prioritized candidate gene for the PHS‐QTL loci *QPhs.wsu‐1A.2*, *QPhs.wsu‐1B.2* and *QPhs1D.1_nwafu*.

We sought to validate our hypothesis that *TaPP2C‐a6* is likely the causal gene for the PHS‐QTL loci *QPhs.wsu‐1A.2*, *QPhs.wsu‐1B.2 and QPhs1D.1_nwafu* with an independent wheat genome‐wide association analysis (GWAS) population consisting of 240 accessions. Our population comprised 240 geographically diverse common wheat cultivars and elite lines from China (Zhu *et al*., 2020). The genomic regions for the coding sequences of *TaPP2C‐a6‐1A* or *1D* with frequent SNP variations were firstly identified with the WheatUnion database (Wang *et al*., 2020a) (Figures S2 and S3) and then amplified for our 240 wheat accessions (Figure S4). In exon 1 of *TaPP2C‐a6‐1A*, four SNPs (Chr 1A: 571988799, Chr 1A: 571988865, Chr 1A: 571988961, and Chr 1A: 571989062) were identified in our GWAS population resulting in six haplotypes (designated as A1 to A6; Figure 1g), while in exon 3 of *TaPP2C‐a6‐1D*, another four SNPs (Chr 1D: 476208465, Chr 1D: 476208433, Chr 1D: 476208353, and Chr 1D: 476208305) were found leading to four haplotypes (designated as D1 to D4; Figure 1h). We performed 4‐year field experiments, obtained the PHS phenotypes and revealed that the *TaPP2C‐a6‐1A* haplotype A2 consistently had higher PHS germination rates than those of the other haplotypes (Figure 1i and Figure S5). In addition, the *TaPP2C‐a6‐1D* haplotype D4 exhibited higher PHS germination rates than those of the other haplotypes (Figure 1j). Collectively, our results demonstrated that *TaPP2C‐a6* haplotypes are associated with PHS phenotypes and may serve as the target gene for improving wheat PHS resistance.

### Both TaPP2C‐a6 and TaPP2C‐a7 are involved in ABA signalling, but TaPP2C‐a6 has stronger interactions with TaDOG1Ls than TaPP2C‐a7

**Figure 2:**
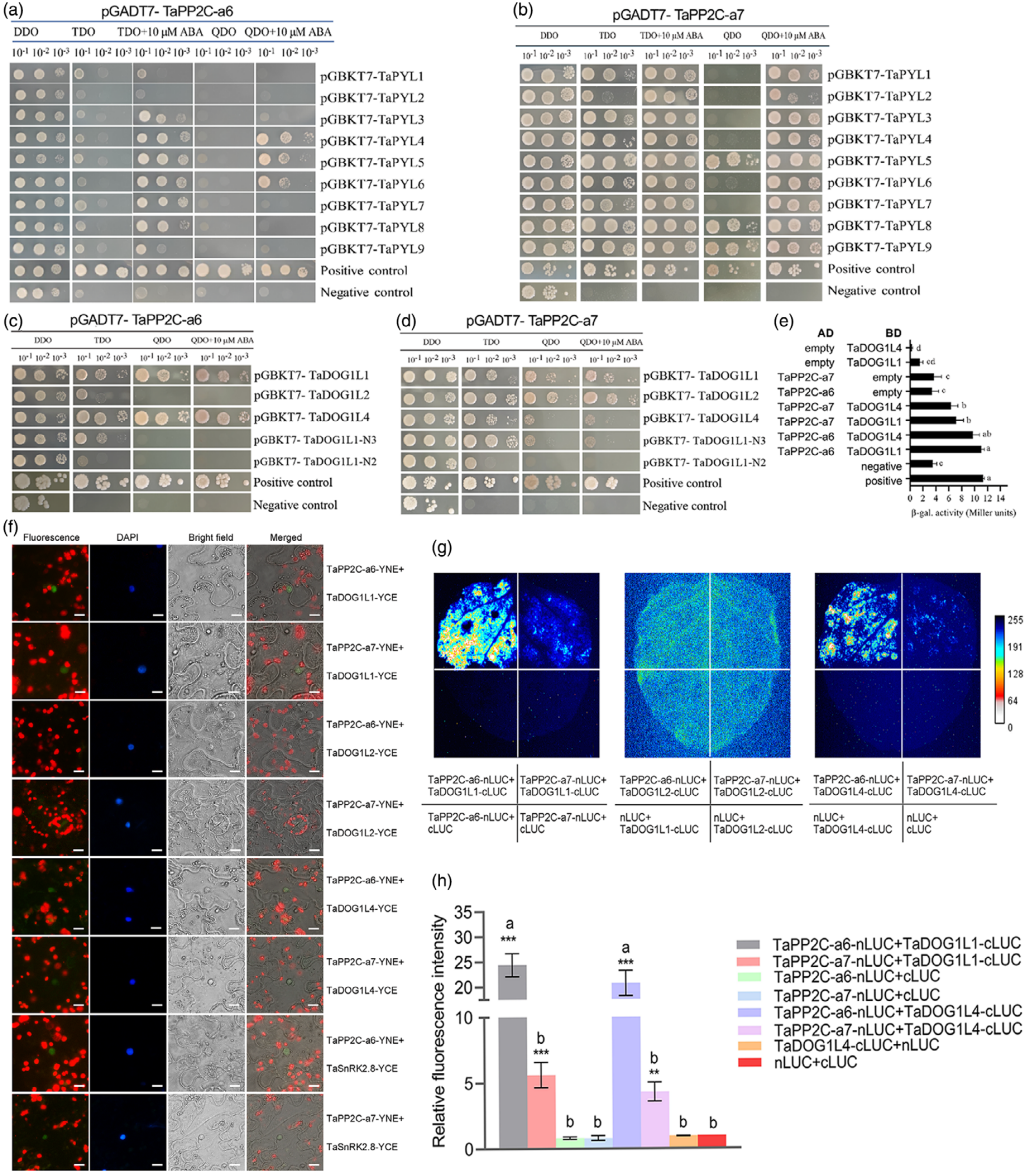
Protein–protein interactions analyses demonstrated that both TaPP2C‐a6 and TaPP2C‐a7 involve in ABA signalling and TaPP2C‐a6 had strong interaction intensity with TaDOG1L1/L4 than that of TaPP2C‐a7. (a) The interactions of pGADT7‐TaPP2C‐a6 and pGBKT7‐TaPYLs. (b) The interactions of pGADT7‐TaPP2C‐a7 and pGBKT7‐TaPYLs. (c) The interactions of pGADT7‐TaPP2C‐a6 and pGBKT7‐TaDOG1Ls. (d) The interactions of pGADT7‐TaPP2C‐a7 and pGBKT7‐TaDOG1Ls. The Y2H experiments were repeated three times (with similar results), and representative images are presented. (e) The β‐galactosidase assay quantified the interaction intensities between TaPP2C‐a6‐TaDOG1Ls or TaPP2C‐a7‐TaDOG1Ls. Data are presented as means ± SEM of three biological replicates. Statistical differences of the interaction intensity were determined with ANOVA by using the SPSS software, labelled using the letter presentation (*post‐hoc* Tukey's test, *P* < 0.05). (f) The BiFC assay confirmed the interactions between TaPP2C‐a6/a7 and TaDOG1Ls or TaSnRK2.8, respectively. Scale bar represents 20 μm. (g) The LCA imaging showed that TaPP2C‐a6 had stronger interaction with TaDOG1L1 and TaDOG1L4 than TaPP2C‐a7's. The BiFC and LCA experiments were performed in triplicates with similar results observed, and the representative images are presented here. (h) The relative fluorescence intensity of LCA assays were used to quantify and compare the interaction intensities of TaPP2C‐a6‐TaDOG1Ls and TaPP2C‐a7‐TaDOG1Ls. At least six leaves were observed for each group. Statistical differences of fluorescence intensity between the LCA experimental groups were calculated with ANOVA by using the SPSS software and are given with letter presentation (*post‐hoc* Tukey's test). The asterisks indicate significant differences of the interaction intensity for each LCA experimental group when compared with the control (determined by Student's *t*‐test; ** and *** standing for *P* < 0.01 and *P* < 0.001, respectively).

We first sought to validate TaPP2C‐a6's involvement in ABA signalling and its interactions with TaDOG1 family members to characterize the function of TaPP2C‐a6 in wheat seed dormancy and germination. *TaPP2C‐a7*, which is highly expressed during seed development, was also studied. Since clade‐A PP2Cs are known to be negative regulators of ABA signalling (Fuchs *et al*., 2013), protein–protein interactions between TaPP2C‐a6, TaPP2C‐a7, and ABA‐signalling core components were investigated using yeast two‐hybrid (Y2H), including nine ABA receptor proteins (TaPYL1, 2, 3, 4, 5, 6, 7, 8, and 9) (Mao *et al*., 2022; Mega *et al*., 2019) and 10 TaSnRK2 proteins (TaSnRK2.1, 2.2, 2.3, 2.4, 2.5, 2.6, 2.7, 2.8, 2.9, and 2.10) (Figure 2a,b; Figure S6a,b). In the presence of ABA (10 μM), TaPP2C‐a6 interacted with TaPYL4/5/6 (Figure 2a), whereas TaPP2C‐a7 interacted with all TaPYLs (Figure 2b). The Y2H showed that both TaPP2C‐a6 and TaPP2C‐a7 specifically interacted with class III TaSnRK2.8, and bimolecular fluorescence complementation (BiFC) experiments further confirmed the interactions between them, but not with other class I and II TaSnRK2s members (Figure 2f; Figure S6a,b). Class III SnRK2 homologues in Arabidopsis (AtSnRK2.2, AtSnRK2.3, and AtSnRK2.6) play essential roles in ABA signalling‐mediated seed development and dormancy (Nakashima *et al*., 2009). These results demonstrated that TaPP2C‐a6 and TaPP2C‐a7 are involved in the ABA‐signalling pathway. Additionally, a subcellular localization assay showed that TaPP2C‐a6 is located in the cytoplasm and nucleus (Figure S6c), which is consistent with its function in the ABA‐signalling transduction.

Ectopic expression analysis of *TaDOG1Ls* in Arabidopsis has demonstrated that TaDOG1L1 and TaDOG1L4 are likely the major DOG1L members that control seed dormancy and germination (Ashikawa *et al*., 2010, 2013). Thus, the interactions between TaPP2C‐a6, TaPP2C‐a7, and the TaDOG1 family members were examined. TaPP2C‐a6 interacted with TaDOG1L1 and TaDOG1L4, whereas TaPP2C‐a7 interacted with TaDOG1L1/2/4 and TaDOG1L‐N3 in the Y2H cells (Figure 2c,d). In addition, BiFC experiments further demonstrated that both TaPP2C‐a6 and a7 interacted with TaDOG1L1 and TaDOG1L4. However, we did not observe any interaction between TaPP2C‐a7 and TaDOG1L2 in the BiFC assay (Figure 2f). Among the TaDOG1 family, *TaDOG1L1* was primarily expressed during seed maturation and plays a major role in wheat seed dormancy (Ashikawa *et al*., 2014; Yu *et al*., 2020). More importantly, β‐galactosidase and split‐luciferase complementation assay (LCA) revealed that TaPP2C‐a6 interacted more strongly with TaDOG1L1 and TaDOG1L4 than those of TaPP2C‐a7 (Figure 2e,g,h). These results indicated that TaPP2C‐a6 and TaPP2C‐a7 are AHG1 TaPP2Cs involved in ABA signalling and dormancy in wheat seeds, and TaPP2C‐a6 may play a more important role than TaPP2C‐a7 in seed dormancy.

### TaPP2C‐a6 overexpression in Arabidopsis leads to a more severe reduction in ABA sensitivity than that of TaPP2C‐a7

**Figure 3:**
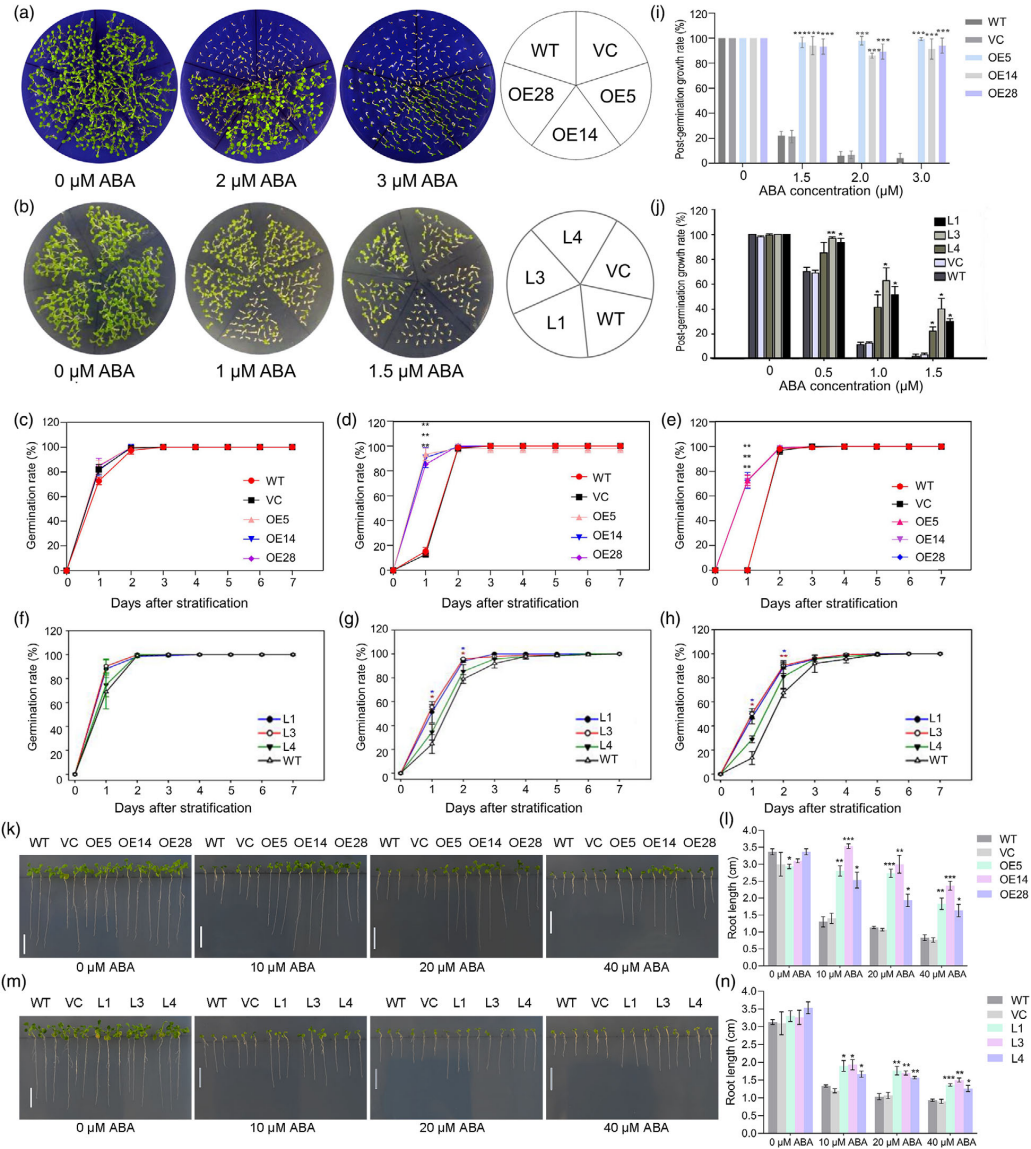
Phenotypic comparison suggests that *TaPP2C‐a6* overexpression in Arabidopsis has stronger impacts on the ABA signalling and insensitivity than *TaPP2C‐a7* overexpression. (a, b) The germination phenotypes of *TaPP2C‐a6* (a) and *TaPP2C‐a7* (b) transgenic plants as well as the vector control (VC) and wild‐type (WT) plants. Seeds were grown on 1/2 MS plates containing various concentrations of ABA for 7 days. The germination experiments were repeated three times with similar results, and representative images are presented. (c–e, i) Germination (c–e) and post‐germination growth (i) rates of seeds from *TaPP2C‐a6* transgenic lines, VC and WT plants in the presence of 0 μM (c), 2 μM (d) and 3 μM (e) ABA after stratification for 7 days. (f–h, j) Germination (f–h) and post‐germination growth (j) efficiencies of seeds from *TaPP2C‐a7* transgenic lines and WT plants in the presence of 0 μM (f), 1 μM (g) and 1.5 μM (h) ABA after stratification for 7 days. The germination and post‐germination growth rate data are shown as means ± SEM of three biological replicates. At least 50 seeds were observed in each replicate per line. The asterisks indicate significant differences compared with the WT (Student's *t*‐test, *P < 0.05; **P < 0.01). (k, m) Phenotypes of root growth of the *TaPP2C‐a6* (k) and *TaPP2C‐a7* (m) transgenic lines, as well as the VC and WT plants. For the *TaPP2C‐a6/a7* transgenic plants, 5‐day‐old seedlings from 1/2 MS plates were transferred to 1/2 MS with 0, 10, 20 or 40 μM ABA for 7 days to examine the root growth phenotypes. The root growth experiments were repeated three times with similar results, and representative images are presented. Each replicate contained three roots. (l, n) Statistical analysis of the primary root lengths of *TaPP2C‐a6* (l) and *TaPP2C‐a7* (n) transgenic lines. The root length data are presented as means ± S.E.M. of three biological replicates. The asterisks indicate significant differences compared with WT. (Student's *t*‐test, *P < 0.05, **P < 0.01, ***P < 0.001). Scale bar represents 1 cm.

Transgenic lines constitutively overexpressing either *TaPP2C‐a6* or *TaPP2C‐a7* (namely ‘*a6*‐OE’ and ‘*a7*‐L’ lines hereafter) were generated in Arabidopsis to study the functions of TaPP2C‐a6 or TaPP2C‐a7 in ABA signalling and seed dormancy and germination. Three homozygous lines were screened for *a6*‐OE (*a6*‐OE5, *a6*‐OE14, and *a6*‐OE28) and *a7*‐L (*a7*‐L1, *a7*‐L3, and *a7*‐L4) transgenic events (Figure S7). Both *a6*‐OE and *a7*‐L Arabidopsis lines showed reduced ABA sensitivity (Figure 3a,b), whereas *TaPP2C‐a6* overexpression lines exhibited more severe ABA‐insensitive phenotypes. For example, the germination rates of the *a6*‐OE lines could reach ~100% at high concentrations of ABA (up to 3 μM) in 2 days after seed stratification (Figure 3c–e). In contrast, the seed germination was affected in the *a7*‐L lines at a lower ABA concentration (1.5 μM) (Figure 3f–h), even though the germination rates of both *a6*‐OE and *a7*‐L lines were significantly higher than those of the controls. Besides, the growth rates of *TaPP2C‐a6* overexpression lines were higher than those of the *TaPP2C‐a7* overexpression lines after seed germination, with the *a6*‐OE lines having a higher than 80% growth rate under 3 μM ABA treatment (Figure 3i,j). ABA inhibits the growth of primary roots in Arabidopsis. We then tested whether the overexpression of *TaPP2C‐a6* and *TaPP2C‐a7* could reduce ABA sensitivity and promote primary root growth. Indeed, both *a6*‐OE and *a7*‐L lines of Arabidopsis could maintain root growth compared with the wild‐type under 10, 20, and 40 μM ABA treatments. *TaPP2C‐a6* overexpression lines exhibited more severe ABA‐insensitive primary root growth than those of *TaPP2C‐a7* overexpression lines (Figure 3k–n).

In addition, changes in the expression of representative ABA‐responsive genes in the *a6*‐OE and *a7*‐L lines under normal growth conditions were analysed using qRT‐PCR. The expression levels of ABA‐responsive genes in the *a6*‐OE lines, including class III *SnRK2.2*, *SnRK2.3*, *SnRK2.6*, *ABI3* (Guerriero *et al*., 2009), *ABI4* (Shu *et al*., 2013), and *ABI5* (Hu *et al*., 2019), dramatically decreased to different degrees compared with those in the WT (Figure S8). In contrast, the expression of *ABI5* and *RD29B* decreased in *TaPP2C‐a7‐*L lines (Figure S9). The phenotypic and molecular analyses results demonstrated that TaPP2C‐a6 and TaPP2C‐a7 can act as negative regulators of the ABA signalling pathway in Arabidopsis.

### Overexpression of TaPP2C‐a6, not TaPP2C‐a7, leads to pre‐harvest sprouting in Arabidopsis

**Figure 4:**
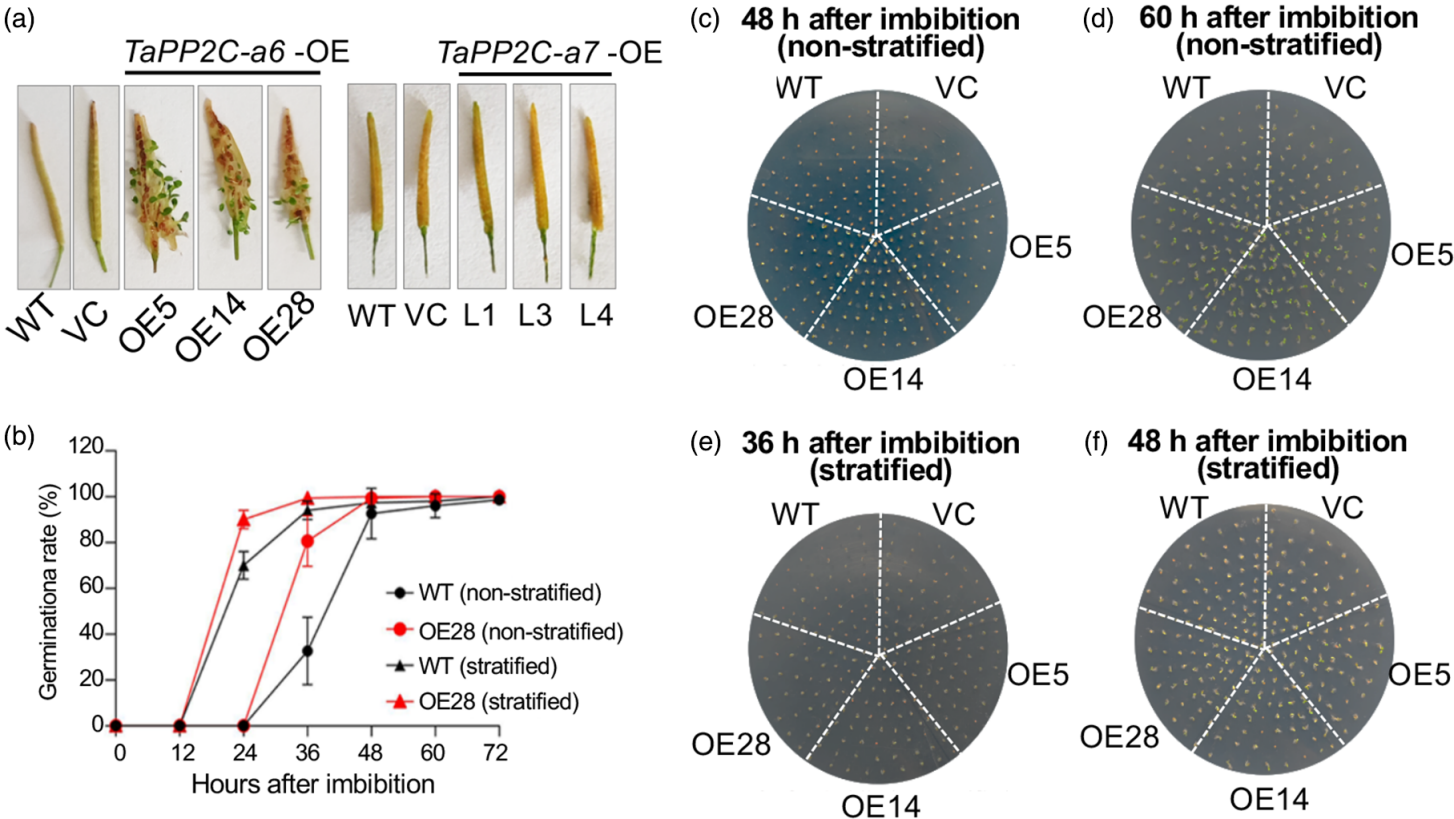
The overexpression of *TaPP2C‐a6*, not *TaPP2C‐a7*, leads to pre‐harvest sprouting in Arabidopsis. (a) Germination of siliques at 18 DAF after incubation for 5 days at 100% humidity using plates with water‐saturated filter paper. The PHS experiments were repeated three times with similar results, and representative images are presented. At least eight siliques were observed in each replicate per line. Scale bar represents 0.5 cm. (b) Time courses showing seed germination rates of *TaPP2C‐a6* transgenic (red lines) and WT (black lines) after non‐stratification (circles) or stratification for 4 days (triangles). The germination rate data are presented as means ± SEM of three biological replicates. (c, d) Germination of non‐stratified seeds from mature siliques uniformly harvested at 30 DAF at 1/2 MS plates after imbibition for 48 h and 60 h, respectively. (e, f) Germination of stratified seeds from mature siliques uniformly harvested at 30 DAF at 1/2 MS plates after imbibition for 36 h and 48 h, respectively. The dormancy experiments were repeated three times with similar results, and representative images are presented. At least 50 seeds were observed in each replicate per line.

Based on the seed‐ and germination‐specific expression patterns of *TaPP2C‐a6* and the interactions between TaPP2C‐a6 and TaDOG1Ls, we investigated whether *TaPP2C‐a6* overexpression affects seed dormancy in Arabidopsis. Firstly, the germination experiments using siliques (harvested 18 days after flowering, DAF) from the transgenic and non‐transgenic lines demonstrated that the overexpression of *TaPP2C‐a6*, not *TaPP2C‐a7*, led to an obvious viviparous phenotype (Figure 4a). Second, we assessed the germination rates of seeds uniformly harvested from mature siliques at 30 DAF from WT, VC, and *TaPP2C‐a6* transgenic Arabidopsis plants with or without stratification. The seeds of the three *a6*‐OE lines germinated more rapidly than those of WT and VC lines without stratification (Figure 4c,d). After stratification for 4 days (to break seed dormancy for consistent and rapid germination), the seed germination of *a6*‐OE lines was almost the same as that of WT and VC lines (Figure 4e,f). The germination rates over 72 h were plotted for the WT and a representative *a6*‐OE line (OE28), showing that *TaPP2C‐a6* overexpression caused rapid germination without stratification (Figure 4b). These results indicated that TaPP2C‐a6 acts as a positive regulator of seed germination and that an increase in its expression level reduces the seed dormancy level.

### TaPP2C‐a6 modulates ABA sensitivity and seed germination in wheat

**Figure 5:**
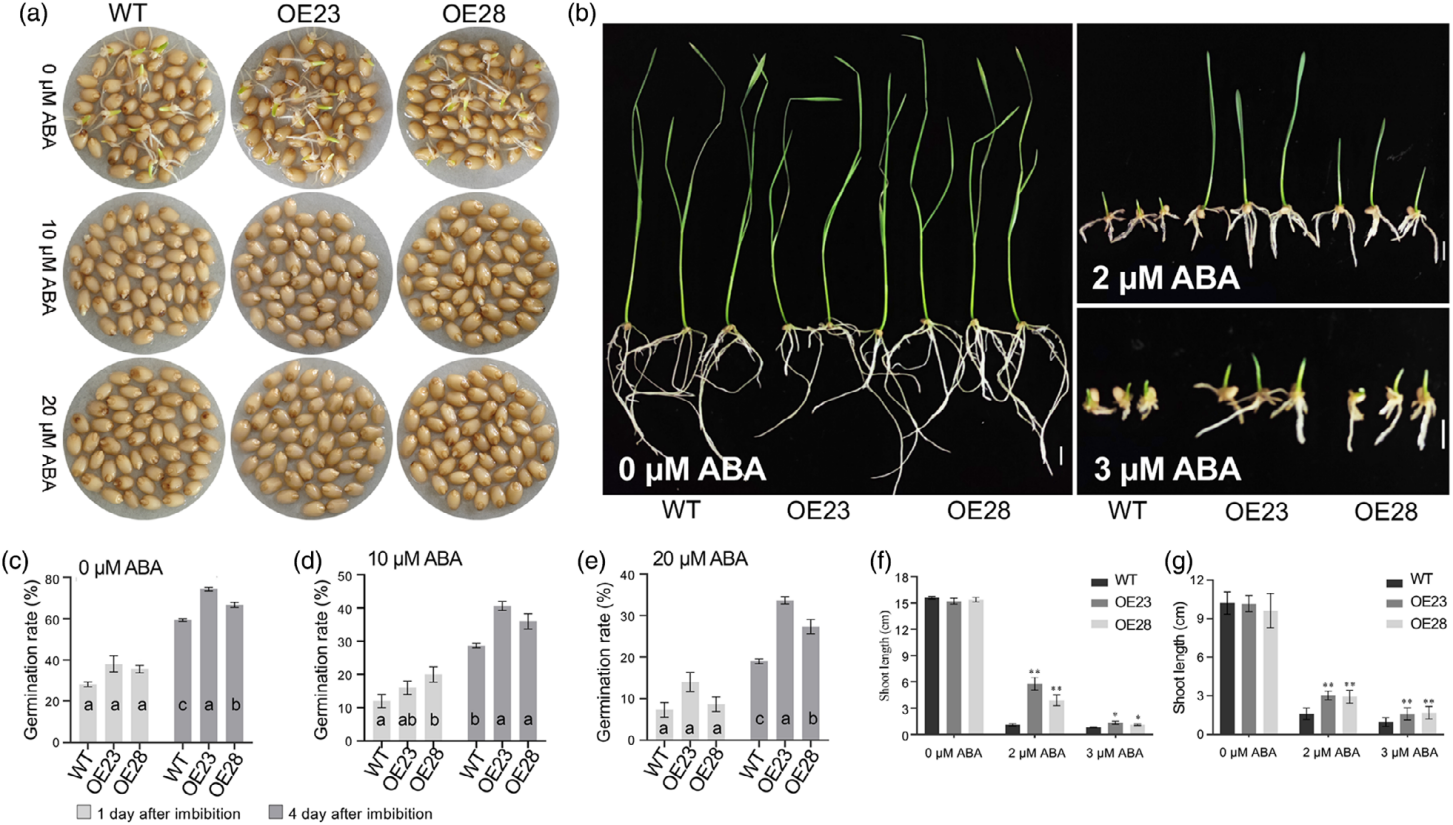
*TaPP2C‐a6* overexpression in wheat promotes seed germination and seedling growth under ABA treatments. (a) Phenotypes of seed germination of *TaPP2C‐a6* OE and WT lines after application for 0, 10, and 20 μM ABA treatments for 2 days. The germination experiments were repeated three times with similar results, and representative images are presented. (b) Phenotypes of shoots and roots of *TaPP2C‐a6* OE and WT lines treated with 0, 2, and 3 μM ABA for 10 days. The experiments were repeated three times with similar results, and representative images are presented. Scale bar represents 1 cm. (c–e) Seed germination tests of *TaPP2C‐a6* OE and WT lines with the presence of 0 (c), 10 (d) or 20 (e) μM ABA, respectively. The germination rate data at 1 day or 4 days after seed imbibition are presented as means ± SEM of three biological replicates. At least 50 seeds were observed in each replicate per line. Statistical differences in the germination rate were determined by ANOVA followed by *post‐hoc* Tukey's test (*P* < 0.05). (f, g) Comparisons between of the shoot (f) and root (g) lengths of *TaPP2C‐a6* OE and WT lines, respectively. Shoot and root lengths were measured after uniformed germinated seeds of *TaPP2C‐a6* OE and WT lines were transferred to MS medium containing 0, 2 and 3 μM ABA for 10 days. Data are presented as means ± SEM of three biological replicates. At least 3 seedlings were observed in each replicate in per line. The asterisks indicate significant differences compared with WT. (Student's *t*‐test, *P < 0.05, **P < 0.01).

**Figure 6:**
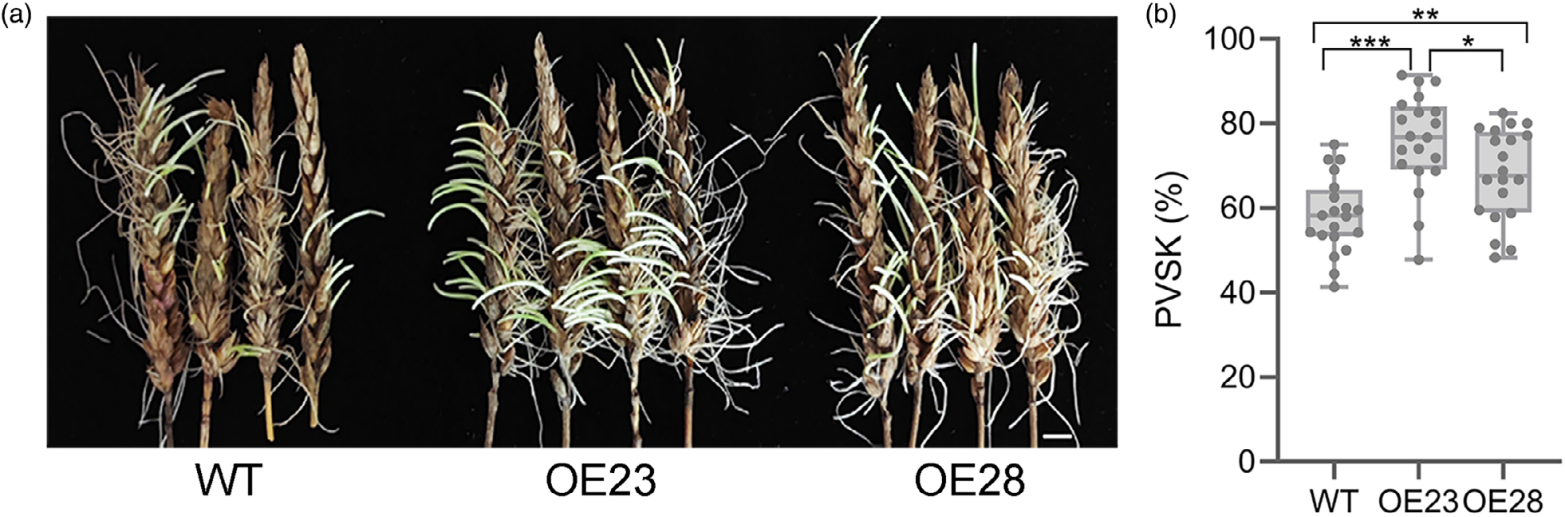
*TaPP2C‐a6* overexpression promotes seed germination in wheat. (a) Representative wheat spikes of WT and *TaPP2C‐a6* OE23 and OE28 plants show a higher level of pre‐harvest sprouting in the *TaPP2C‐a6* overexpression lines than in WT. The photograph was taken after they were kept in a moist chamber for 5 days at 22 °C. The PHS experiments were repeated three times with similar results, and representative images are presented. The scale bar represents 1 cm. (b) Box plots summarize the percentage of visible sprouted kernels (PVSK) from 20 replicates for each of the WT and *TaPP2C‐a6* OE23 and OE28 plants. Lines in the box plots indicate the median. The significant differences in PVSK values were determined with the student's *t*‐test (*P < 0.05, **P < 0.01, ***P < 0.001).

Several independent transgenic lines overexpressing *TaPP2C‐a6* were generated in wheat to determine whether TaPP2C‐a6 also regulates seed dormancy and germination. Quantitative RT‐PCR results showed that *TaPP2C‐a6* had higher expression levels in *TaPP2C‐a6*‐OE plants than in the non‐transgenic controls (*cv*. L88‐31) (Figure S10). Two lines of *TaPP2C‐a6*‐OE (i.e. OE23 and OE28) were further characterized. We first determined the effects of *TaPP2C‐a6* overexpression on seed germination and ABA signalling. Under normal conditions, *TaPP2C‐a6*‐OE lines germinated more rapidly than non‐transgenic WT seeds, with significantly higher germination rates observed in the *TaPP2C‐a6‐*OE lines (more than 66% versus 59.3% for the WT; Figure 5a,c). The germination rates of WT significantly decreased to less than 20% with the increase of ABA concentrations (from 10 μM to 20 μM) during germination, while the seeds from the *TaPP2C‐a6*‐OE lines reached a germination rate of ~30% (33.7% and 27.3% for OE23 and OE28, respectively) after 4‐day imbibition (Figure 5a,d,e). This demonstrated that *TaPP2C‐a6* overexpression likely reduces ABA sensitivity and partly overcomes the ABA‐mediated inhibition of seed germination. We evaluated whether *TaPP2C‐a6* overexpression could relieve ABA‐mediated inhibition of growth. Without ABA treatment, the seedling growth of *TaPP2C‐a6*‐OE lines was similar to that of the non‐transgenic control (Figure 5b). However, under ABA (2 μM to 3 μM) treatments, shoot and root growth were inhibited for the germinated seeds. In contrast, the growth of *TaPP2C‐a6*‐OE lines was inhibited to a much lesser extent (Figure 5b,f,g; Figure S11). The negative regulators of ABA signalling induced by ABA may serve as desensitization mechanisms to promote the continued growth of plants under stress conditions (Miao *et al*., 2020; Wang *et al*., 2019). These data suggested that *TaPP2C‐a6* negatively regulates ABA signalling in wheat and that ABA hyposensitization promotes seedling growth under ABA treatment conditions. More importantly, we measured the PHS of these transgenic wheat lines as the percentage of visible sprouted kernels (PVSK) per spike (Figure 6a). The *TaPP2C‐a6*‐OE23 line exhibited a PVSK of ~47.8%–91.4%, while the OE28 line had a PVSK of ~48.3%–82.5%. In contrast, the non‐transgenic line (WT) showed a PVSK of ~41.4%–71.4%, which was significantly lower than the PVSK values of the *TaPP2C‐a6*‐OE lines (Figure 6b). Hence, *TaPP2C‐a6* overexpression strongly decreased PHS tolerance in wheat. Additionally, The *TaPP2C‐a6*‐OE23 line had a higher PVSK value than that of the *TaPP2C‐a6*‐OE28 line, correlating with the difference in *TaPP2C‐a6* expression levels between the two transgenic wheat lines (Figure S10). This suggested that TaPP2C‐a6 might regulate pre‐harvest sprouting in a dosage‐dependent manner. The results demonstrated that the expression level of *TaPP2C‐a6* was associated with a decrease in ABA sensitivity and PHS resistance, supporting its role as a negative regulator of wheat PHS resistance.

## Discussion

PHS has become a global issue for cereal production and food security. Current research and breeding applications on improving PHS resistance in wheat have relied mainly on QTL mapping, while the causal genes for most of the PHS‐QTL intervals have not been cloned, hindering not only our mechanistic understanding of PHS resistance but also precise breeding to target the causal genes of PHS (Tai *et al*., 2021). Up to date, only a handful of genes have been known to regulate seed dormancy in wheat, including *TaMYB10* (Himi *et al*., 2002, 2011), *TaAFP* (Feng *et al*., 2019), *TaSdr* (Zhang *et al*., 2017), *TaJAZ1* (Ju *et al*., 2019), *TaQsd1* (Abe *et al*., 2019), *TaMFT‐3A* (Nakamura *et al*., 2011), *TaMKK3‐A* (Torada *et al*., 2016), *TaPI4K‐2A* (Tai *et al*., 2024) and *TaVp‐1* (Yang *et al*., 2007); not to say that only some of these functionally validated PHS genes have been investigated for population genetics and breeding purposes (Chang *et al*., 2023; Yiwen *et al*., 2022). Therefore, the functional characterization of the genes involved in seed dormancy and germination of wheat will not only provide more target genes for the improvement of PHS resistance but, more importantly, will enhance our insights into the regulatory network underlying this biological process.

In contrast to the limited knowledge of seed dormancy and germination in wheat, numerous efforts have been made for uncovering the molecular mechanisms in model species (such as Arabidopsis, rice and maize), in which the DOG1‐mediated and ABA signalling‐mediated pathways represent two major regulatory modules (Tai *et al*., 2021). For the ABA signalling‐mediated pathway, many ABA‐signalling core components have been found to play a role in seed dormancy. On one hand, the active SnRK2s phosphorylate and activate the bZIP transcription factor ABI5, which regulates the expression of ABA‐responsive genes via binding the ABRE element at the promoter regions. This is an important mechanism of Arabidopsis seed dormancy (Carles *et al*., 2002; Nakashima *et al*., 2009; Umezawa *et al*., 2010). On the other hand, seed germination requires PP2Cs, which bind and inactivate SnRK2s, thereby reducing the ABA sensitivity of seeds (Nishimura *et al*., 2007; Umezawa *et al*., 2009). *AHG1* (*ABA‐hypersensitive germination 1*) and *AHG3* are most highly expressed in dry seed among nine members of the clade‐A *PP2C* genes, negatively regulating the ABA‐inhibited seed germination (Nishimura *et al*., 2007; Yoshida *et al*., 2006). Recently, DOG1 was found to be able to interact with AHG1/AHG3, inhibit the PP2C activity, and thus suppress seed germination (Nee *et al*., 2017a; Nishimura *et al*., 2018). This brings together the ABA‐ and DOG1‐mediated dormancy pathways in PP2C and represents a major breakthrough in the study of the molecular mechanism of seed dormancy. Although significant progress has been made in the modes of action of important dormancy regulators in dormancy induction and maintenance in recent years, it is still unknown how seeds transit from the dormancy stage to the germination stage.

In the present study, we compared the effects of overexpression of *TaPP2C‐a6* with *TaPP2C‐a7*. Interestingly, TaPP2C‐a6 was able to significantly decrease seed dormancy level, leading to the PHS phenotype in both Arabidopsis and wheat, whereas TaPP2C‐a7 did not affect the dormancy level of transgenic Arabidopsis plants (Figures 4, 5a,c and 6a). We found that the interaction intensity between TaPP2C‐a6 and TaDOG1Ls was significantly higher than that of TaPP2C‐a7 and TaDOG1Ls (Figure 2e,g,h). Based on the likely conserved role of TaDOG1Ls and AtDOG1 in seed dormancy (Ashikawa *et al*., 2010, 2013; Carrillo‐Barral *et al*., 2020), we hypothesized that TaPP2C‐a6 might also have a stronger interaction with AtDOG1 than that of TaPP2C‐a7. Such a hypothesis could explain the fact that heterologous expression of *TaPP2C‐a6*, but not *TaPP2C‐a7*, affects the seed dormancy levels in Arabidopsis. In wheat, our results reveal that the TaPP2C‐a6‐TaDOG1Ls interaction intensity could be a new regulatory aspect involved in the TaDOG1Ls‐ and ABA‐mediated control of seed dormancy. These results suggest that PP2Cs‐DOG1Ls might be a possible regulatory nexus of seed dormancy conserved from dicots to monocots. It is worth stressing that the interactions between clade‐A PP2Cs and DOG1Ls within a crop species or between crops and Arabidopsis merit further investigation to hopefully gain new insights into whether such potential PP2Cs‐DOG1Ls regulation could be evolutionarily conserved. In wheat, *TaPP2C‐a6* was specifically expressed during seed maturity, while *TaPP2C‐a7* was broadly expressed and had a relatively higher expression level in leaves compared with other tissues (Figure 1c, Figures S1b,c and S12a). *TaDOG1L1* and *TaDOG1L4* had similar expression patterns with *TaPP2C‐a6* (Yu *et al*., 2020). Besides, *TaPP2C‐a6* was up‐regulated with a higher expression level than that of *TaPP2C‐a7* with or without the ABA treatment during seed imbibition (Figure 1e,f). In rice, *OsABIL2* and *OsABIL2* ^*G183D*^ overexpression resulted in PHS and reduced ABA sensitivity of rice seeds (Endo *et al*., 2018; Li *et al*., 2015). *OsPP2C51*, the closest homologue of *TaPP2C‐a6* (Figures S1a and S12b,c), was also predominantly expressed in the embryo of mature seed, which was different from the expression pattern of other clade‐A *OsPP2C* genes, and positively regulated rice seed germination. Overexpression of *OsPP2C51* leads to increased expression levels of α‐amylase synthesizing genes, acting as downstream effectors in seed germination signalling (Bhatnagar *et al*., 2017). In a word, the spatiotemporal expression specificity and TaPP2C‐TaDOG1L interaction intensity may both contribute to the TaPP2C‐a6‐mediated regulation on seed dormancy in wheat, pinpointing *TaPP2C‐a6* as the likely causal gene for the PHS‐QTL (i.e. *QPhs.wsu‐1A.2/1B.2* and *QPhs1D.1_nwafu*).

Also, we employed population genetic approaches to further substantiate *TaPP2C‐a6* as the likely causal gene for several reported PHS‐QTLs. We admit that the unavailability of the original mapping population for the PHS‐QTL *QPhs.wsu‐1A.2/1B.2* hindered us from directly showing that it is the causal gene (Martinez *et al*., 2018). Alternatively, we sought to investigate whether *TaPP2C‐a6* genotypes (or haplotypes) could be associated with PHS phenotypes in independent wheat populations. As shown by our results, in the 240 representative Chinese wheat cultivars and elite lines, haplotypes of *TaPP2C‐a6‐1A* are indeed associated with higher PHS germination rates, supporting our claim that *TaPP2C‐a6* is the likely causal gene of PHS‐QTLs. Furthermore, another independent GWAS study using a different a wheat population identifies the PHS‐QTL *QPhs1D.1_nwafu* and suggests *TaPP2C‐a6‐1D* as the candidate gene within this QTL interval (Tai *et al*., 2024). Owing to the limited time and costs, the target genomic regions of *TaPP2C‐a6* containing frequent non‐synonymous SNPs were PCR amplified (Wang *et al*., 2020a) and the gene‐based genotype–phenotype association analysis was performed (Figure 1g–j, Figures S2–S5). However, several limitations should be worthy of noting: (1) the GWAS population used in our study comprises representative modern wheat cultivars and elite lines, of which the seed dormancy might be directly or indirectly selected to allow rapid and uniform germination (with median PHS germination rates more than 60% in most environments; Figure 1i,j), thus not suitable for mining superior *TaPP2C‐a6* alleles that confer low PHS germination rates; (2) PCR and Sanger sequencing can reveal parts of the sequence polymorphisms of *TaPP2C‐a6*, and therefore future studies with whole genome or exome resequencing approaches are recommended to fully discover the genetic diversity and superior alleles of *TaPP2C‐a6* to support wheat molecular breeding of PHS resistance. Nevertheless, our results showed that several *TaPP2C‐a6* haplotypes (e.g. *TaPP2C‐a6‐A2* and *TaPP2C‐a6‐D4*) are associated with high PHS germination rates and may be used to screen out those germplasm with low PHS resistance. More investigations regarding the exploration of superior *TaPP2C‐a6* alleles conferring better PHS tolerance from landraces and/or wild relatives are needed. In addition, our transgenic results demonstrated that *TaPP2C‐a6* overexpression weakens PHS resistance in wheat. Knock‐down or editing of *TaPP2C‐a6* with CRISPR‐based techniques merits future studies to obtain deeper molecular insights into *TaPP2C‐a6*'s regulation of seed dormancy in wheat. Notably, considering the expression redundancy between the *TaPP2C‐a6* homoeologs, editing of all three *TaPP2C‐a6* homoeologs might be necessary for future genetic studies.

**Figure 7:**
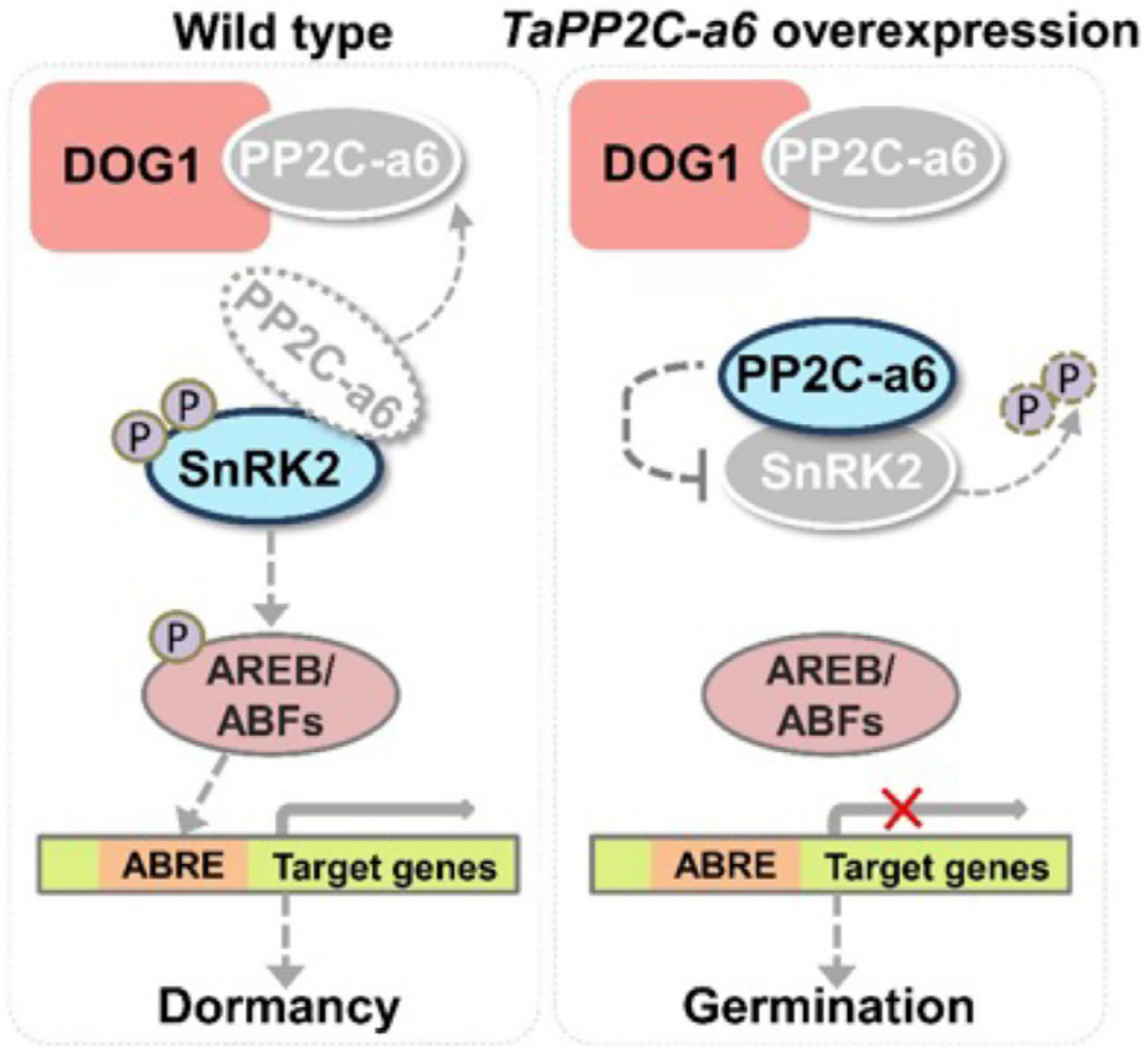
The schematic model illustrating the proposed regulatory mechanism of seed dormancy and germination by TaPP2C‐a6. In the wild‐type wheat cultivar (i.e. L88‐31), TaPP2C‐a6 interacted with TaSnRK2s and could inhibit their kinase activities, while TaDOG1Ls strongly interacted with TaPP2C‐a6 and could suppress the TaPP2C‐a6‐TaSnRK2 interactions, possibly causing the release of the TaSnRK2 kinase activity to activate the downstream AREB/ABFs transcription factors. In the model species, AREB/ABFs activate ABA‐responsive genes by binding to the ABA‐responsive elements in their promoters, which in turn activates the ABA signalling during seed development to promote seed dormancy. In *TaPP2C‐a6* overexpression wheat lines, our results supported that higher levels of TaPP2C‐a6 likely interact with TaDOG1Ls and TaSnRK2s, and the TaPP2C‐a6‐TaSnRK2 interactions could inhibit the kinase activities of TaSnRK2s, which attenuated ABA signalling to promote seed germination.

In conclusion, our study demonstrates that TaPP2C‐a6 regulates seed dormancy and germination, thus affecting wheat PHS resistance. The association between *TaPP2C‐a6* haplotypes and PHS phenotypes, together with other GWAS results (Martinez *et al*., 2018; Tai *et al*., 2024), supports *TaPP2C‐a6* as the likely causal gene of several PHS‐QTLs with breeding potential to improve PHS resistance in wheat. In parallel to the genetic data, TaPP2C‐a6‐TaDOG1Ls interaction intensity could be a new regulatory aspect to fine‐tune seed dormancy in wheat. Considering the potentially conserved roles of DOG1 between Arabidopsis and wheat and our molecular analysis between TaPP2C‐a6 and TaDOG1Ls, the hypothetical model of TaPP2C‐a6‐mediated regulation of seed dormancy in wheat is given in Figure 7. One possible explanation of increased germination caused by *TaPP2C‐a6* overexpression could be that abundant TaPP2C‐a6 proteins suppress SnRK2s and downstream ABA responses during seed maturation, thus attenuating ABA‐signalling‐mediated seed dormancy. It is worth noting that the role of TaDOG1Ls in TaPP2C‐a6‐mediated control of seed dormancy deserves future investigations. Nevertheless, our work serves as a proof‐of‐concept that combining PHS‐QTLs and the genes related to the ABA signalling pathway could be a valuable reverse genetic approach to efficiently identify causal genes for numerous wheat PHS‐QTLs, facilitating our understanding of seed dormancy and germination and molecular breeding for improving PHS resistance. It will be interesting to study whether this reverse genetic approach may work for GA‐related genes to uncover more causal genes of PHS‐QTL regions, as existing studies in rice and wheat have demonstrated GA‐related genes are either the causal genes or involved in the regulation of PHS (reviewed in Tai *et al*., 2021).

## Experimental procedures

### Plant materials and growth conditions

The common wheat (*Triticum aestivum* L.) cultivar L88‐31 was used for gene cloning, expression analyses, and genetic transformation (Guan *et al*., 2023). Arabidopsis ecotype Col‐0 was used to generate transgenic Arabidopsis plants expressing *TaPP2Cs*. Arabidopsis seeds were sterilized using standard procedures, sown on 1/2 Murashige and Skoog (MS) medium, and transferred to a greenhouse (25 °C, 16 h light/8 h dark) for cultivation. Arabidopsis seedlings were grown with a mixture of soil:vermiculite (3:1 ratio) at 22 °C with relative humidity of 60%–70% in the growth chamber (16 h light/8 h dark). Wheat seeds were sterilized with a 75% ethanol solution (1 min) followed by a 0.1% HgCl_2_ solution (8 min) and washed at least four times with sterile water.

A natural population comprising 240 wheat lines comprising 229 from 12 provinces in China and 11 accessions introduced from CIMMYT was used in the present study (Table S6). All lines were planted in the experimental field at Nanhu Experimental Station in Wuhan, Hubei province (30°483′ N, 114°316′ E) during four cropping seasons from 2016 to 2019. Field management follows local agricultural practices throughout the growing period.

To measure the PHS resistance of these wheat lines, spikes were harvested at the grain filling stage. Ten intact spikes with 10 cm peduncle were collected from each line and separated into two replicates (5 spikes per technical replicate). All spikes were air‐dried for 7 days at room temperature, avoiding direct sunlight and high temperature, then stored at −20 °C to maintain dormancy until tests were conducted (within 6 weeks).

Spike‐wetting tests were conducted in a rain simulation chamber to evaluate PHS tolerance. Spikes from the freezer were placed upright on perforated stainless‐steel trays and naturally air‐dried at room temperature for 2 days. The rain simulation chamber was set to spray water for 20 min every 4 h at 20 ± 2 °C. During day 2, 0.025 mg/mL Quintozene solution was sprayed for 5 min to control fungal contamination. After 5 days of incubation, each germinated spike was put in a bag and stored at −20 °C until manual counting. Germinated spikes were threshed by hand. Grain was considered germinated when the length of either the radicle or plumule exceeded half of the grain's length. Germination percentage (GP) was calculated as the percentage of germinated grains to the total number of grains in 5 spikes of a line.

### Total RNA extraction and qRT‐PCR

Wheat L88‐31 seedlings were grown in a greenhouse, and samples were collected at the following tissues and stages to profile the expression levels of *TaPP2Cs* across developmental tissues and stages: young roots, stems, and leaves sampled at the three‐leaf stage; mature roots, stems, leaves, flag leaves, pistils, and stamens sampled at the flowering stage; palea, lemma, caryopsis, embryo, and endosperm sampled from mature wheat plants at 20 days post‐anthesis (dpa); and grains sampled at different dpas. The mature seeds were imbibed for 0, 3, 6, 12, 24, 36, and 48 h in solution with or without 30 μM ABA. All samples were immediately frozen using liquid nitrogen and stored at −80 °C for expression analyses.

Total RNA was extracted with the Plant Total RNA Extraction Kit (Zomanbio, China) and reverse transcribed to cDNA with the FastKing gDNA Dispelling RT SuperMix Kit (Tiangen, China) for quantitative reverse transcription‐PCR (qRT‐PCR) analysis. The qRT‐PCR analysis was performed with three biological replicates using a CFX96 Connect Real‐Time PCR System (Bio‐Rad, Hercules, CA) with the AceQ qPCR SYBR Green Master Mix Kit (Vazyme). *TaActin1* (Chinese Spring RefSeqv1.1 *TraesCS1A02G274400*) was used as the reference gene. All the primers used are listed in Table S7. The qRT‐PCR data were analysed with the 2^−ΔΔCT^ method (Livak and Schmittgen, 2001). All qPCR‐based quantification experiments were performed in triplicates.

### Gene cloning and expression analysis

The cDNA of *TaPP2C‐a6‐1D* (Chinese Spring RefSeqv1.1 *TraesCS1D02G419000*) and *TaPP2C‐a7‐3D* (Chinese Spring RefSeqv1.1 *TraesCS3D02G355900*) were cloned and confirmed using Sanger sequencing (Augct Biotech, China), as described previously (Yu *et al*., 2019) (the primers are listed in Table S7). The protein sequences of TaPP2C‐a6‐1D and TaPP2C‐a7‐3D homologues in other plant species were obtained from Ensembl Plants (http://plants.ensembl.org/index.html). MEGA11 software (Kumar *et al*., 2018) was used to perform multiple sequence alignments using MUSCLE, and a neighbour‐joining (NJ) phylogenetic tree was constructed with 1000 bootstrap replicates.

The expression atlas of the wheat cultivar Azhurnaya across multiple developmental stages and tissues and the expression patterns in the embryo and endosperm tissues were retrieved from the WheatOmics database (Ma *et al*., 2021; Ramírez‐González *et al*., 2018; Wei *et al*., 2019) to examine the expression profiles of ABA‐signalling core genes. Differentially expressed genes were determined within each RNA‐seq dataset, according to routine criteria (DEseq‐calculated *q* values < 0.05, and |log_2_(fold change)| > 1) (Li *et al*., 2019).

### Protein subcellular localization analysis

The *TaPP2C‐a6‐1D* coding sequence was amplified (primers are shown in Table S7) and cloned into the pBI121‐GFP vector via homologous recombination using a One Step Cloning Kit (Vazyme, China) to express the TaPP2C‐a6‐1D‐GFP fusion protein under the control of the *CaMV35S* promoter. The empty vector pBI121‐GFP and recombinant pBI121‐TaPP2C‐a6‐1D‐GFP constructs were transiently expressed in tobacco leaf epidermal cells using *Agrobacterium*‐mediated transformation (*Agrobacterium tumefaciens* strain EHA105) to determine the subcellular localization of TaPP2C‐a6‐1D (Luo *et al*., 2017). Green fluorescence signals were detected using an IX71 fluorescence microscope (Olympus, Tokyo, Japan) 2 days after transformation.

### Yeast two‐hybrid (Y2H) assay

*TaDOG1L‐N2‐2A* and *TaDOG1L‐N3‐6A* were cloned from mixed cDNA templates from different wheat tissues, while the *TaDOG1L* genes were cloned as described by Yu *et al*. (2019). Gene cloning of *TaPYL1‐1A*, *TaPYL2‐3A*, *TaPYL3‐7A*, *TaPYL5‐3B*, *TaPYL6‐1B*, *TaPYL7‐4A*, *TaPYL8‐1B*, and *TaPYL9‐7A* has been reported previously (Mega *et al*., 2019). The coding sequences of *TaPP2C‐a6‐1D* and *TaPP2C‐a7‐3D* were cloned into the pGADT7 vector for the yeast two‐hybrid assay, whereas *TaDOG1Ls* and *TaPYLs* were inserted into the pGBKT7 vector. The plasmids pGBKT7‐TaDOG1L1‐3A, TaDOG1L2‐1D, TaDOG1L4‐3B, pGBKT7‐TaPYL4‐2B, and 10 pGBKT7‐TaSnRK2s plasmids were constructed as reported in previous studies (Yu *et al*., 2019, 2020). All primers used to construct the Y2H constructs are listed in Table S7.

Yeast two‐hybrid assay was performed using the MatchMakerTM Gold Yeast Two‐Hybrid system (Clontech). The yeast strains co‐transformed with the pGADT7‐T and pGBKT7‐p53 vectors and those co‐transformed with the pGADT7‐T and pGBKT7‐Lam vectors were used as positive and negative controls, respectively. A pair of AD and BD constructs was co‐transformed into yeast strain AH109. The positive transformants were first selected by incubation on double‐dropout medium (SD/‐Trp‐Leu, DDO) and then examined in triple‐dropout medium (SD/‐His‐Trp‐Leu, TDO), TDO medium with 10 mM ABA, quadruple‐dropout medium (SD/‐Ade‐His‐Trp‐Leu, QDO), and QDO medium with 10 mM ABA.

β‐Galactosidase analyses were performed according to the manufacturer's instructions (Clontech), using chlorophenol red‐β‐D‐galactopyranoside (CPRG) as the substrate (Zhou *et al*., 2018). Liquid cultures are assayed for β‐galactosidase to quantify the intensity of Y2H‐based protein–protein interactions. One unit of β‐galactosidase is defined as the amount that hydrolyzes 1 μmol of CPRG to chlorophenol red and D‐galactose per minute per cell. β‐galactosidase units = 1000 × OD_578_/(t × V × OD_600_) [t = elapsed time (in minutes) of incubation, V = 0.1 × concentration factor, OD_600_ = A_600_ of 1 mL of culture].

### Bimolecular fluorescence complementation (BiFC) assay

For the BiFC assay, the coding sequences of *TaPP2C‐a6‐1D* and *TaPP2C‐a7‐3D* genes were cloned into the SpYNE vector, while *TaDOG1L2‐1D* and *TaSnRK2.8‐5D* were cloned into the SpYCE vector. SpYCE‐TaDOG1L1‐3A and SpYCE‐TaDOG1L4‐3B plasmids were constructed as reported previously (Yu *et al*., 2020). The SpYCE/SpYNE fusion constructs were transformed into *Agrobacterium tumefaciens* strain EHA105. The 4‐week‐old tobacco leaf epidermal cells were co‐infiltrated with mixtures of equal amounts of SpYCE/SpYNE cultures via *Agrobacterium tumefaciens*‐mediated transient transformation. After 48 h, the fluorescence signals were detected using an 80i fluorescence microscope (Nikon, Japan). Nuclei were stained with 4′,6‐diamidino‐2‐phenylindole (DAPI) dye. All primers used to construct the BiFC constructs are listed in Table S7.

### Split‐luciferase complementation assay (LCA)

The coding sequences of *TaPP2C‐a6‐1D* and *TaPP2C‐a7‐3D* were constructed into the JW771 (N‐terminal half of luciferase, nLUC) vector, whereas *TaDOG1L1*‐3A, *TaDOG1L2*‐1D, and *TaDOG1L4*‐3B were inserted into the JW772 (C‐terminal half of luciferase, cLUC) vector to compare the interactions of TaPP2Cs and TaDOG1Ls. nLUC and cLUC were used as negative controls. The nLUC/cLUC fusion constructs were transformed into *Agrobacterium tumefaciens* GV3101. The 4‐week‐old tobacco leaf epidermal cells were co‐infiltrated with mixtures of equal amounts of nLUC/cLUC cultures via *Agrobacterium tumefaciens*‐mediated transient transformation. To compare the differences in the interaction capacity of TaPP2Cs and TaDOG1Ls, equal amounts of the respective constructs were combined in a 1:1 ratio. Each pair of nLUC/cLUC cultures was injected into one‐quarter of the tobacco leaf area. After 48 h, the leaves were incubated with 1 mM D‐luciferin and potassium salt dissolved in 0.2% (v/v) Triton X‐100 in the dark for 10 min. Fluorescent images were captured using a CCD camera (FluorChem R). The fluorescence images were converted to pseudo‐colour images using the ImageJ software. Relative fluorescence intensity was calculated using ImageJ software. All primers used to generate the LUC constructs are listed in Table S7.

### Haplotype analysis

Total DNA was extracted from the leaves of each variety planted in Hubei in 2023 using the CTAB method for haplotype analysis. Information regarding the *TaPP2C‐a6* SNP was obtained from the WheatUnion database (http://wheat.cau.edu.cn/WheatUnion/b_15/). The gDNA fragments of *TaPP2C‐a6‐1A/1D* were amplified using specific primers. The haplotypes of *TaPP2C‐a6‐1A/1D* were identified using Sanger sequencing (Sangon Biotech, China). Statistical differences between the germination rates of haplotypes were calculated using Welch's *t*‐test using SPSS software.

### Generation of transgenic Arabidopsis plants

*TaPP2C‐a6* and *TaPP2C‐a7* coding sequences were cloned into the pBI121 and pSN1301 vectors, respectively. TaPP2C was ubiquitously overexpressed under the control of the *CaMV35S* promoter (*TaPP2C‐a6‐OE* and *TaPP2C‐a7‐OE*) in both vectors. pBI121‐TaPP2C‐a6 and pBI121, pSN1301‐TaPP2C‐a7, and pSN1301 empty vectors were transformed into Arabidopsis using the floral‐dip method with *Agrobacterium tumefaciens* strain EHA105 (Clough and Bent, 1998). Transgenic seeds were selected on 1/2 MS medium containing 50 mg/L kanamycin or 20 mg/L hygromycin B. Homozygous transgenic lines were identified by PCR detection of *TaPP2C*, GUS staining, and antibiotic selection in consecutive generations. The expression levels of *TaPP2C‐a6* and *TaPP2C‐a7* in independent transgenic lines were analysed using qRT‐PCR. Three homozygous lines of *TaPP2C‐a6‐OE* or *TaPP2C‐a7‐OE* were selected for further experiments.

### Seeds dormancy assay in Arabidopsis

Arabidopsis siliques harvested 18 days after flowering (DAF) were incubated at 100% humidity on plates with water‐saturated filter paper for 5 days to determine the dormancy of freshly harvested Arabidopsis seeds. Mature Arabidopsis seeds were harvested at 30 DAF, surface‐sterilized, and incubated on 1/2 MS plates. The seeds were imbibed at 25 °C for 3 days with or without 4‐day stratification (4 °C). The germinated seeds (i.e. the radicles emergenced) were counted at each of the following time points (0, 12, 24, 36, 48, 60 and 72 h). All experiments were performed in triplicate.

### ABA sensitivity assays in Arabidopsis

Approximately, 50 fully mature and air‐dried Arabidopsis seeds from each transgenic and control line were surface‐sterilized and sown on 1/2 MS plates with or without various concentrations of ABA. Seeds were stratified at 4 °C for 4 days in the dark before incubating the plates in the growth chamber. The number of germinated seeds and post‐germination growth (green cotyledon emergence) was measured daily for 7 days.

Five‐day‐old seedlings grown on 1/2 MS plates were transferred to medium with or without various concentrations of ABA for the root growth assay. The primary root lengths were measured after 7 days.

### Generation of transgenic wheat plants

The *TaPP2C‐a6‐1D* coding sequence fused with a 3 × HA tag was cloned into the pAHC25 vector to drive *TaPP2C‐a6* expression using the maize *Ubiquitin1* promoter. The pAHC25‐TaPP2C‐a6‐1D construct was used to transform the immature wheat embryos (*cv*. L88‐31), following the particle bombardment method (Hu *et al*., 2020). Transgenic wheat lines were identified using PCR analysis after T_0_ selection during the tissue culture step (primers are listed in Table S7), and non‐segregant lines were obtained using propagation with PCR detection among consecutive generations (Wang *et al*., 2014a). Quantitative RT‐PCR was performed to determine the expression levels of both transgenic and endogenous *TaPP2C‐a6‐1D* in the transgenic wheat lines.

### Seedling growth assays of transgenic wheat

Surface‐sterilized seeds were sown on MS plates and subsequently stratified at 4 °C for 2 days in the dark to unify their germination before being incubated in the growth chamber. Uniformly germinated seeds were transferred to MS plates and glass plates with or without various concentrations of ABA after 24 h of incubation in the growth chamber. The root and shoot lengths were measured after a 10‐day incubation. Three independent biological replicates were used for analysis.

### PHS and germination assay of transgenic wheat

Wild‐type (WT) and *TaPP2C‐a6‐1D* OE lines were grown in the experimental field of Huazhong University of Science and Technology in the 2022/23 season, and the spikes were harvested at physiological maturity (characterized by the loss of green colour on the spike) to evaluate PHS rates. The spikes were immersed in distilled water for 3 h and then bundled with wet gauze and placed in a plastic bag (90% humidity) before they were incubated in a moist chamber at 22 °C for 5 days. Germinated and non‐germinated seeds were counted for each spike, and the percentage of visible sprouted kernels (PVSK) was determined using 20 random spikes for each line (Guo *et al*., 2018). Approximately, 50 seeds of the WT and *TaPP2C‐a6‐1D* OE lines were sterilized and sown on sterile filter paper in Petri dishes containing various concentrations of ABA for the seed germination assay. The Petri dishes were subsequently transferred to a growth chamber at 25 °C for 7 days. Seed germination was considered when radicle protrusion was visible. Three independent biological replicates were used for analysis.

### Statistical analysis

All experiments were performed in triplicate unless stated otherwise. Statistically significant differences were calculated with SPSS software using Student's *t*‐test (*P < 0.05, **P < 0.01, ***P < 0.001), ANOVA with post‐hoc Tukey's test (*P* < 0.05), or Welch's *t*‐test (*P < 0.05, **P < 0.01, ***P < 0.001).

## Author contributions

G.H., G.Y., and Y.L. conceived the study; Q.Z., X.Y., and L.C. performed the experiments and data analysis; J.C., Y.W., and M.T. were involved in data analysis; R.W., Y.W., and F.S. conducted data acquisition and molecular biology experiments; Y.Z. and H.Z. conducted phenotype analysis and genetic transformation; H.X. and J.P. performed expression analyses and managed materials; G.H. and Z.Z. managed materials and were involved in resource acquisition; Q.Z. and Y.L. wrote the draft manuscript; L.C., G.Y., G.H., M.C., and Y.L. revised the manuscript.

## Conflict of interest

The authors declare no conflict of interest.

## Supporting information


**Figure S1** Phylogenetic analysis of the clade‐A PP2Cs and tissue‐specific expression patterns of *TaPP2C‐a6*.
**Figure S2** SnpFreq analysis results identified the genomic regions of *TaPP2C‐a6* where frequent SNP variations were detected.
**Figure S3** Sequence alignment of *TaPP2C‐a6‐1A*, *1B*, and *1D* genomic DNA.
**Figure S4** Images of agarose gels of PCR amplification products of *TaPP2C‐a6* gDNA in diverse wheat varieties.
**Figure S5** Haplotype analysis of *TaPP2C‐a6* gene.
**Figure S6** The yeast two‐hybrid analysis of TaPP2C‐a6/a7 and TaSnRK2s and the subcellular localization analysis of TaPP2C‐a6.
**Figure S7** Quantification of the *TaPP2C‐a6/a7* expression levels among the Arabidopsis transgenic lines.
**Figure S8** Expression analysis of the ABA‐responsive genes in *TaPP2C‐a6* overexpression and WT lines of Arabidopsis.
**Figure S9** Expression analysis of the ABA‐responsive genes in *TaPP2C‐a7* overexpression and WT lines of Arabidopsis.
**Figure S10** Quantification of the *TaPP2C‐a6* expression levels among the transgenic wheat lines.
**Figure S11** *TaPP2C‐a6* overexpression in wheat reduces the ABA sensitivity during root growth.
**Figure S12** Tissue‐specific expression and sequences identity analysis of *TaPP2C‐a6* and *TaPP2C‐a7*.

**Table S1** Genes encoding the core components of ABA signalling in wheat.
**Table S2** GeneIDs within the linked region of PHS‐QTL *QPhs.wsu‐1A.2*.
**Table S3** GeneIDs within the linked region of PHS‐QTL *QPhs.wsu‐1B.2*.
**Table S4** DEGs identified in wheat seed and embryo samples that are located within the PHS‐QTL *QPhs.wsu‐1A.2*.
**Table S5** DEGs identified in wheat seed and embryo samples that are located within the PHS‐QTL *QPhs.wsu‐1B.2*.
**Table S6** Information of the 240 wheat accessions used in the *TaPP2C‐a6* haplotype analysis.
**Table S7** Primers used in the present study.

## Data Availability

The data that supports the findings of this study are available in the supplementary material of this article.
