# Supplementary material for: TaPP2C‐a6 interacts with TaDOG1Ls and regulates seed dormancy and germination in wheat
Source: Plant Biotechnol J. 2025 May 26;23(8):3313–29. doi: 10.1111/pbi.70144 (PMC12310832; doi:10.1111/pbi.70144)
Supplement: Supplementary file 1 — Figure S1 Phylogenetic analysis of the clade‐A PP2Cs and tissue‐specific expression patterns of TaPP2C‐a6. Figure S2 SnpFreq analysis results identified the genomic regions of TaPP2C‐a6 where frequent SNP variations were detected. Figure S3 Sequence alignment of TaPP2C‐a6‐1A, 1B, and 1D genomic DNA. Figure S4 Images of agarose gels of PCR amplification products of TaPP2C‐a6 gDNA in diverse wheat varieties. Figure S5 Haplotype analysis of TaPP2C‐a6 gene. Figure S6 The yeast two‐hybrid analysis of TaPP2C‐a6/a7 and TaSnRK2s and the subcellular localization analysis of TaPP2C‐a6. Figure S7 Quantification of the TaPP2C‐a6/a7 expression levels among the Arabidopsis transgenic lines. Figure S8 Expression analysis of the ABA‐responsive genes in TaPP2C‐a6 overexpression and WT lines of Arabidopsis. Figure S9 Expression analysis of the ABA‐responsive genes in TaPP2C‐a7 overexpression and WT lines of Arabidopsis. Figure S10 Quantification of the TaPP2C‐a6 expression levels among the transgenic wheat lines. Figure S11 TaPP2C‐a6 overexpression in wheat reduces the ABA sensitivity during root growth. Figure S12 Tissue‐specific expression and sequences identity analysis of TaPP2C‐a6 and TaPP2C‐a7. [file PBI-23-3313-s002.docx]

Supporting Information for

**TaPP2C-a6 interacts with TaDOG1Ls and regulates seed dormancy and germination in wheat**

Qian Zhang ^1†^, Xiaofen Yu^2†^, Ruibin Wang^1^, Ya’nan Wu^1^, Fu Shi^1^, Yufan Zhang^1^, Hongyan Zhao^1^, Huazhen Xu^1^, Jiao Pan^1^, Yuesheng Wang^1^, Min Tu^3^, Junli Chang^1^, Zhanwang Zhu^4,5,6^, Guangyuan He^1,*^, Mingjie Chen^1,*^, Ling Chen^4,5,6*^,Guangxiao Yang^1,*^, Yin Li^1,*^

^1^ The Genetic Engineering International Cooperation Base of Chinese Ministry of Science and Technology, Key Laboratory of Molecular Biophysics of Chinese Ministry of Education, College of Life Science and Technology, Huazhong University of Science and Technology, Wuhan 430074, China;

^2^ Key Laboratory of Plant Germplasm Enhancement and Specialty Agriculture, Wuhan Botanical Garden, Innovative Academy of Seed Design, Chinese Academy of Sciences, Wuhan 430074, China.

^3^ Hubei Technical Engineering Research Center for Chemical Utilization and Engineering Development of Agricultural and Byproduct Resources, School of Chemical and Environmental Engineering, Wuhan Polytechnic University, Wuhan 430023, China

^4^ Institute of Food Crops, Hubei Academy of Agricultural Sciences, Wuhan 430064, China

^5^ Hubei Key Laboratory of Food Crop Germplasm and Genetic Improvement, Wuhan 430064, China

^6^ Key Laboratory of Crop Molecular Breeding, Ministry of Agriculture and Rural Affairs, Wuhan 430064, China

*Corresponding authors: G.H. (hegy@hust.edu.cn); M.C. (cmj@hust.edu.cn); L.C. (lingchen@hbaas.com); G.Y. (ygx@hust.edu.cn); Y.L. (yinli2021@hust.edu.cn)

This file contains the following supporting information.

**Figure S1.** Phylogenetic analysis of the clade-A PP2Cs and tissue-specific expression patterns of *TaPP2C-a6*.

**Figure S2.** SnpFreq analysis results identified the genomic regions of *TaPP2C-a6* where frequent SNP variations were detected.

Figure S3. Sequence alignment of *TaPP2C-a6-1A*, *1B* and *1D* genomic DNA.

Figure S4. Images of agarose gels of PCR amplification products of *TaPP2C-a6* gDNA in diverse wheat varieties.

Figure S5. Haplotype analysis of *TaPP2C-a6* gene.

**Figure S6.** The yeast two-hybrid analysis of TaPP2C-a6/a7 and TaSnRK2s and the subcellular localization analysis of TaPP2C-a6.

**Figure S7.** Quantification of the *TaPP2C-a6/a7* expression levels among the Arabidopsis transgenic lines.

**Figure S8.** Expression analysis of the ABA-responsive genes in *TaPP2C-a6* overexpression and WT lines of Arabidopsis.

**Figure S9.** Expression analysis of the ABA-responsive genes in *TaPP2C-a7* overexpression and WT lines of Arabidopsis.

**Figure S10.** Quantification of the *TaPP2C-a6* expression levels among the transgenic wheat lines.

**Figure S11.** *TaPP2C-a6* overexpression in wheat reduces the ABA sensitivity during root growth.

**Figure S12.** Tissue-specific expression and sequences identity analysis of *TaPP2C-a6* and *TaPP2C-a7*.

**Table S1.** Genes encoding the core components of ABA signaling in wheat.

**Table S2.** GeneIDs within the linked region of PHS QTL *QPhs.wsu-1A.2*.

**Table S3.** GeneIDs within the linked region of PHS QTL *QPhs.wsu-1B.2*.

**Table S4.** DEGs identified in wheat seed and embryo samples that are located within the PHS QTL *QPhs.wsu-1A.2*.

**Table S5.** DEGs identified in wheat seed and embryo samples that are located within the PHS QTL *QPhs.wsu-1B.2*.

**Table S6.** Information of the 240 wheat accessions used in the *TaPP2C-a6* haplotype analysis.

**Table S7.** Primers used in the present study.


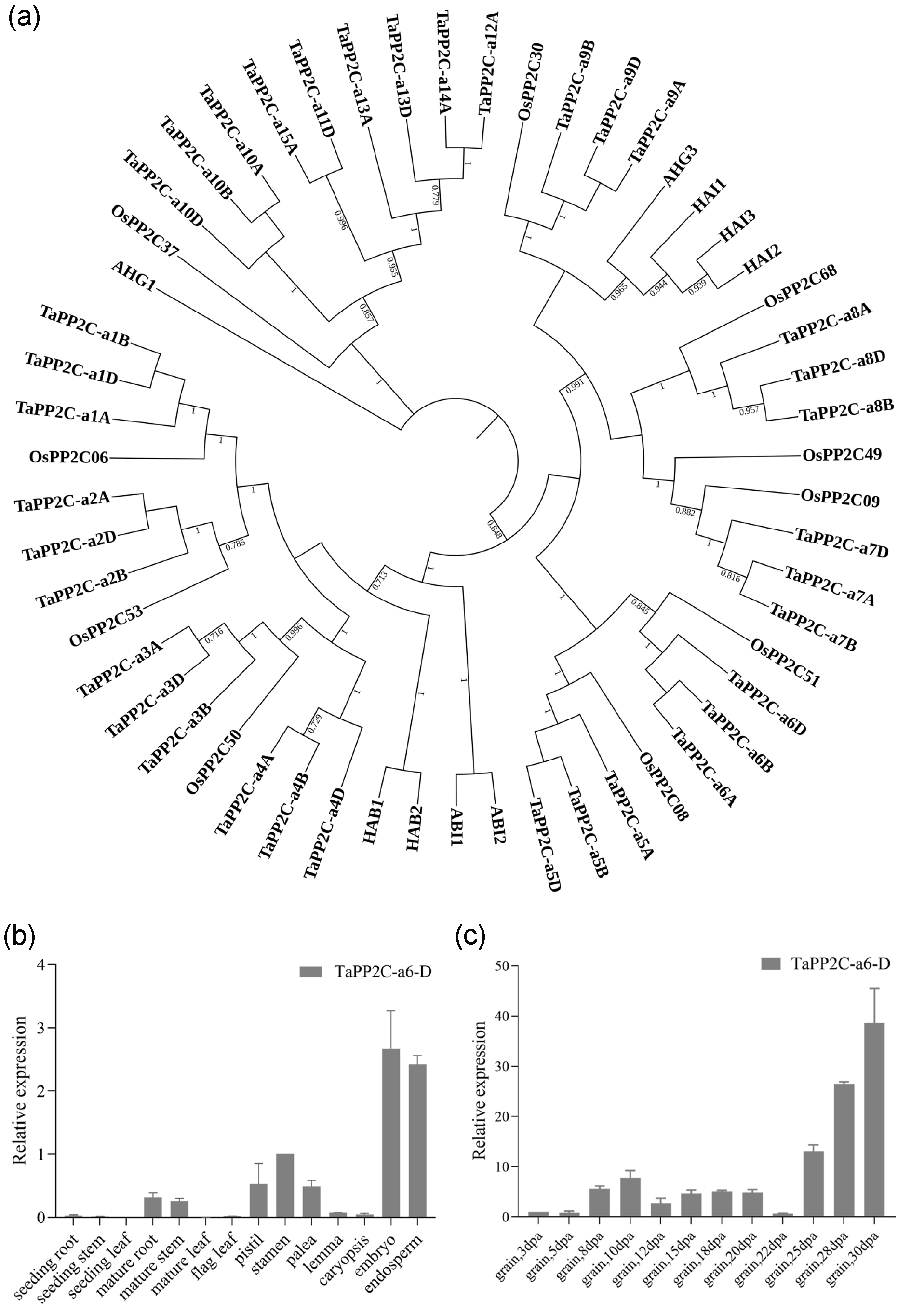


Figure S1. Phylogenetic analysis of the clade-A *PP2Cs* and tissue-specific expression patterns of *TaPP2C-a6*. (a) Phylogenetic tree of clade-A *PP2Cs* from Arabidopsis thaliana, *Oryza sativa* and *Triticum aestivum* (cultivar Chinese Spring) constructed by using the neighbor-joining method with 1000 bootstraps with the MEGA11 software. (b) Tissue-specific expression analysis of *TaPP2C-a6* determined by using the qRT-PCR method. (c) Quantitative RT-PCR-based expression analysis of *TaPP2C-a6* in a series of developmental stages of wheat grains, ranging from 3 days post anthesis (dpa) to 30 dpa. *TaActin* was used as the internal reference gene. Data are presented as means ± standard error of the mean (S.E.M.) for three biological replicates.


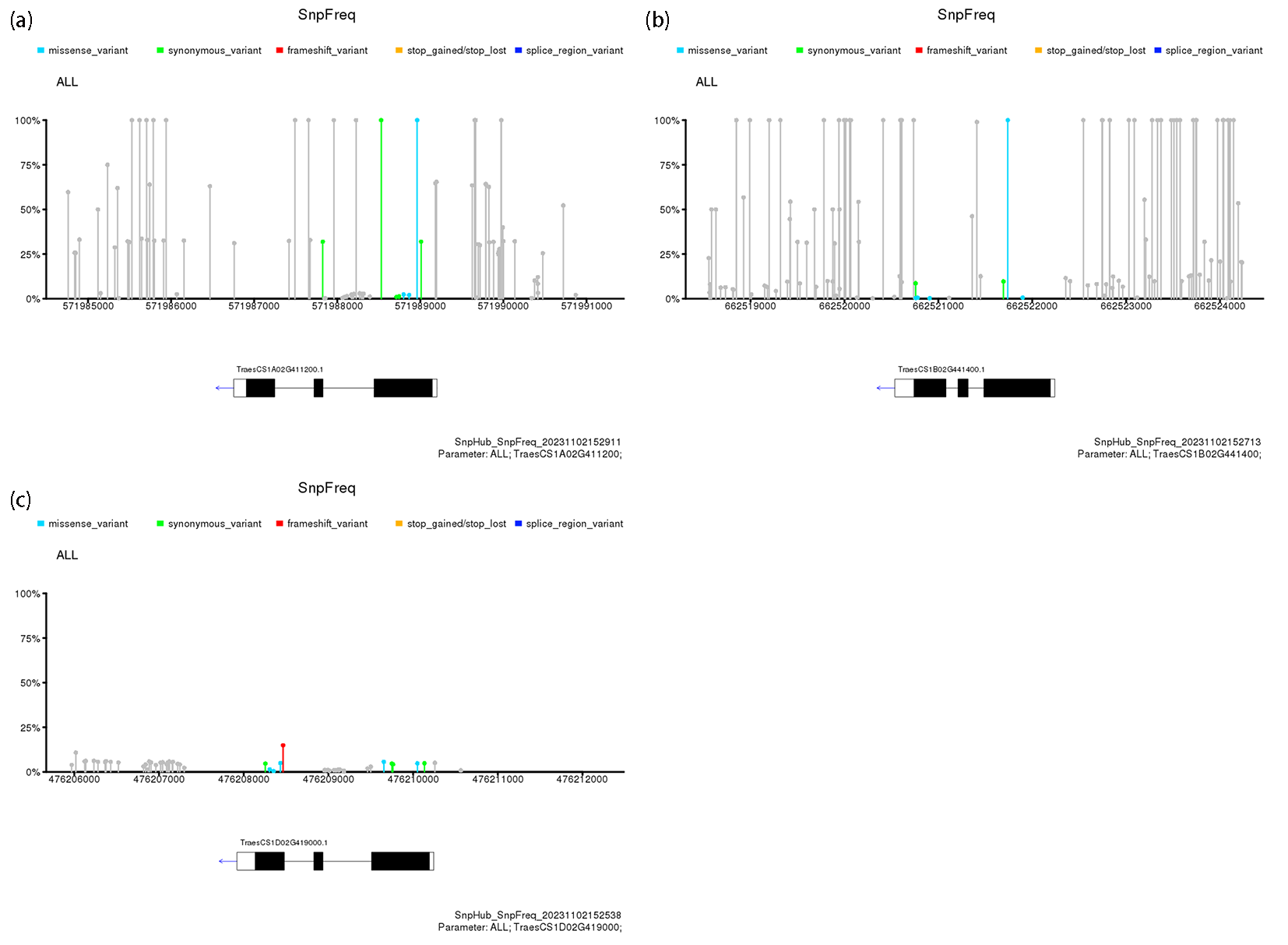


Figure S2. SnpFreq analysis results identified the genomic regions of *TaPP2C-a6* where frequent SNP variations were detected. (a) The SnpFreq analysis result of *TaPP2C-a6-1A*. (b) The SnpFreq analysis result of *TaPP2C-a6-1B*. (c) The SnpFreq analysis result of *TaPP2C-a6-1D*.


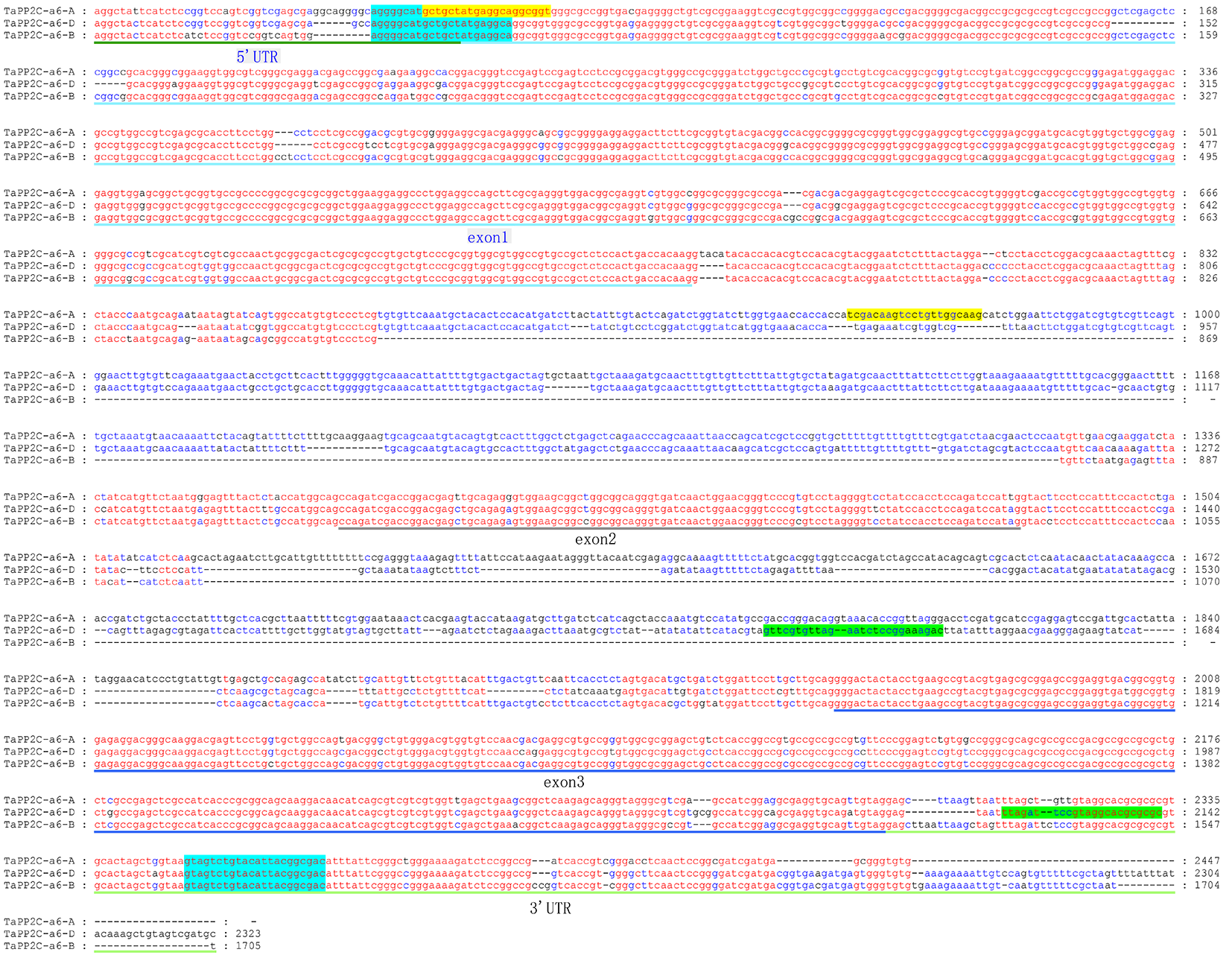


Figure S3. Sequence alignment of the *TaPP2C-a6-1A*, *1B* and *1D* genomic DNA (gDNA). The three exons of *TaPP2C-a6-1A*, *1B* and *1D*, respectively, are highlighted underline. The invariant and conserved nucleotides are colored in red and blue, respectively. The prime pairs used to specifically amplify the gDNA fragment of *TaPP2C-a6-1A*, which contains several nucleotides showing variants among wheat accessions, is highlighted in yellow shade. The prime pairs used to specifically amplify the gDNA fragment of *TaPP2C-a6-1D*, which contains several nucleotides showing variants among wheat accessions, is highlighted in green shade. The light-blue shade indicates the primer pairs used to amplify the cDNA of *TaPP2C-a6*.


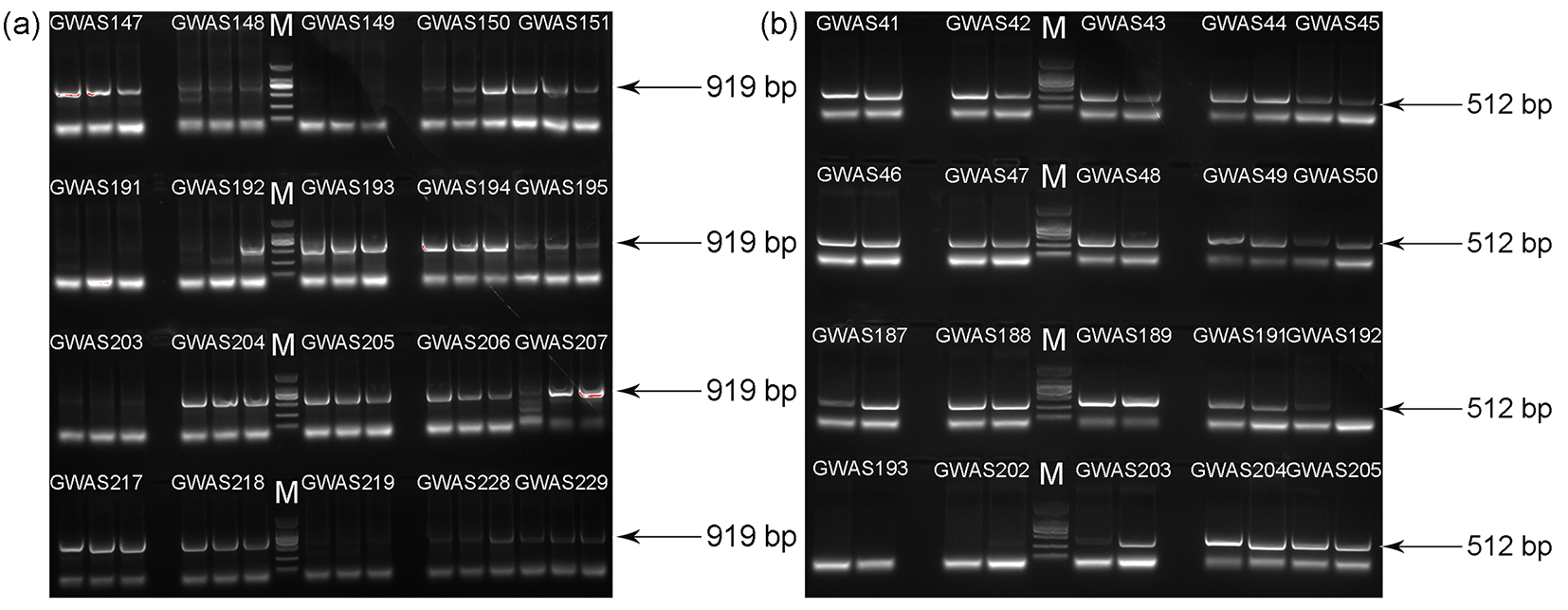


Figure S4. Images of agarose gels of PCR amplification products of *TaPP2C-a6* gDNA in diverse wheat varieties. (**a**, **b**) Images of agarose gels of PCR amplification products of *TaPP2C-a6-1A* (**a**) and *1D* (**b**) gDNA in diverse wheat varieties.


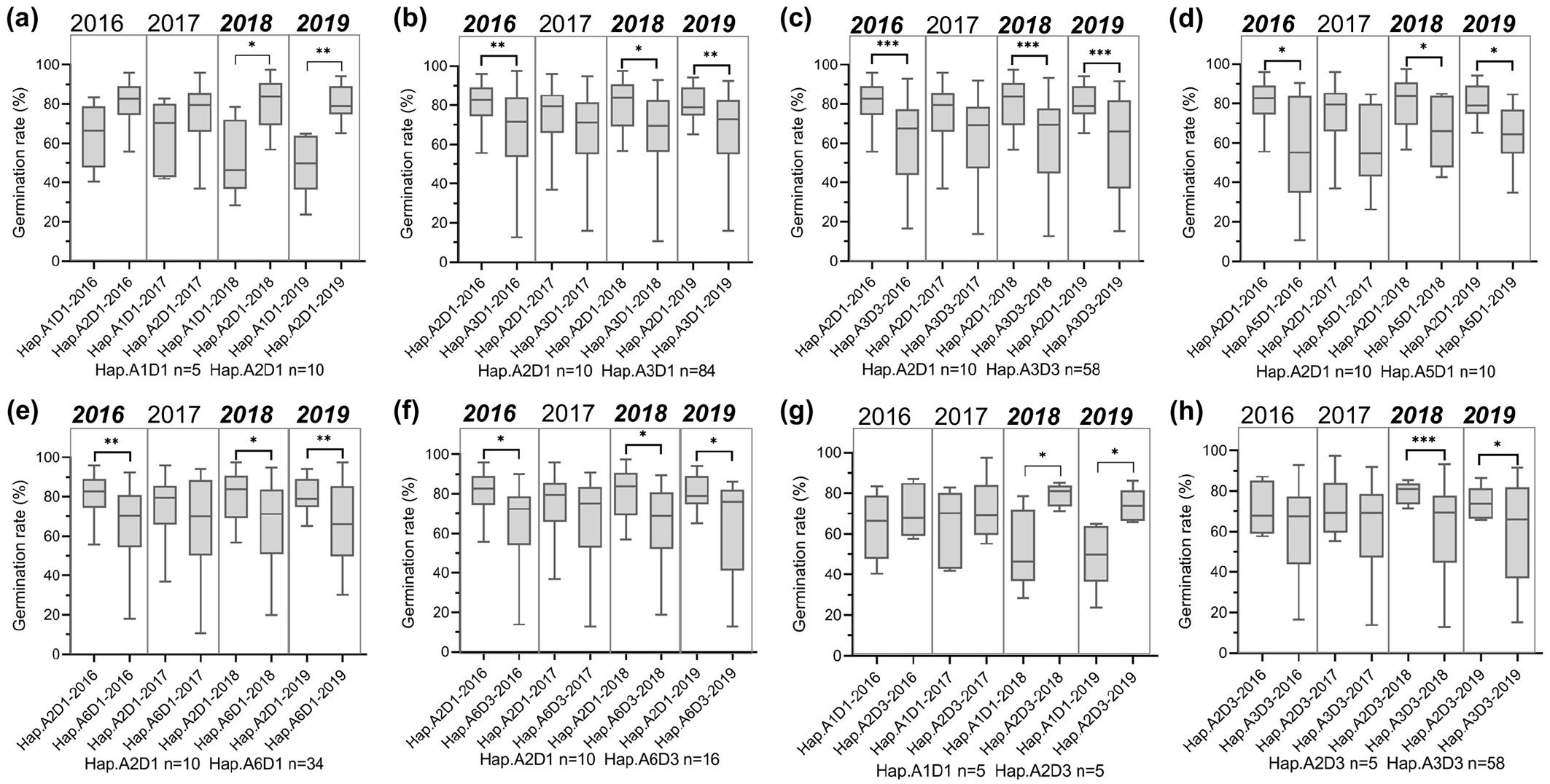


Figure S5. Haplotype analysis of *TaPP2C-a6* gene. (a-h) Boxplots for germination rates based on the haplotypes for *TaPP2C-a6-1A* and *1D* in 2016 to 2019. Box edges represent the 0.25 quantile and 0.75 quantile with the median values shown by bold lines. Differences between the haplotypes were analyzed by Welch’s *t*-test. (*, ** and *** standing for *P*<0.05, *P*<0.01 and *P*<0.001, respectively).


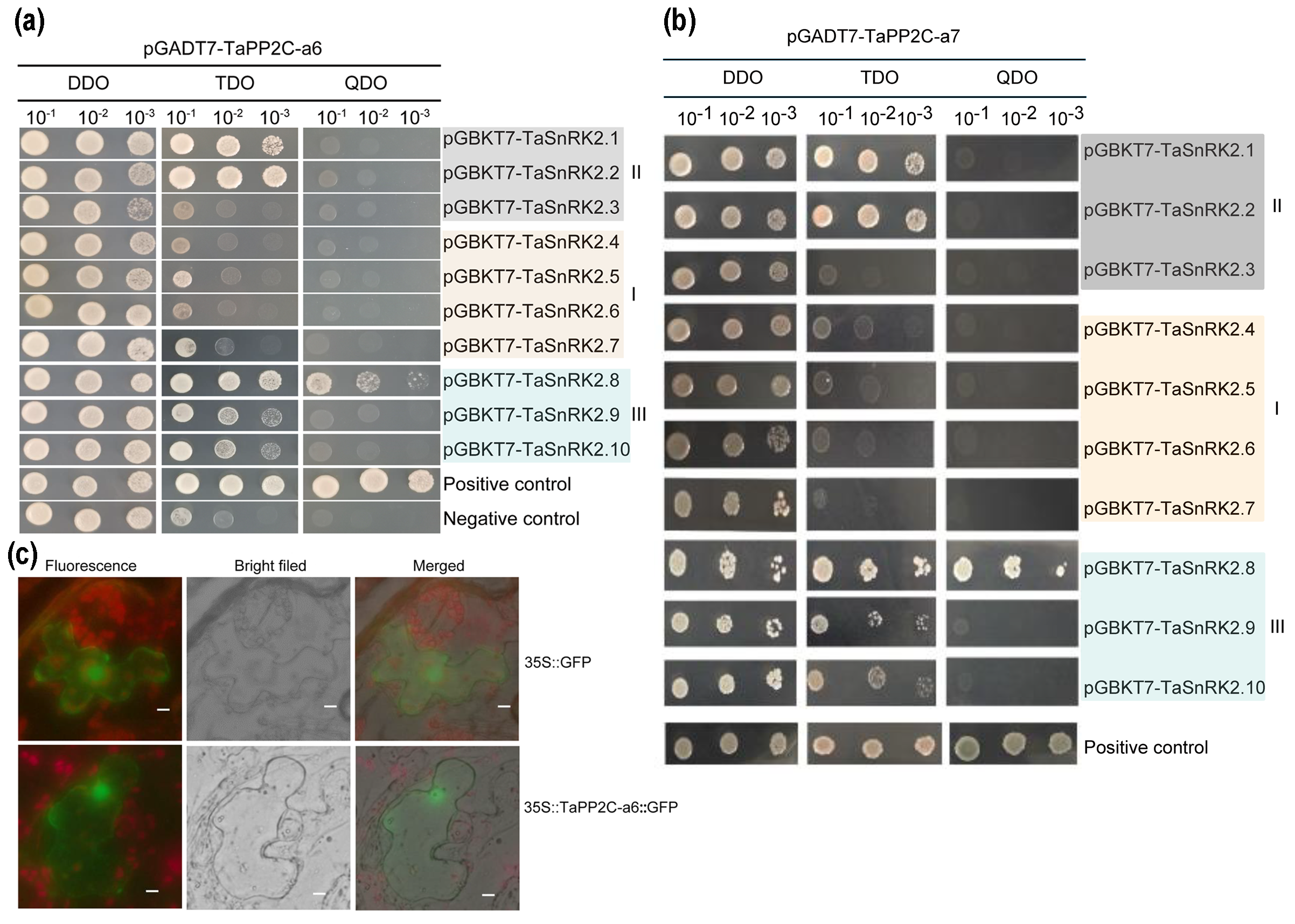


**Figure S6.** The yeast two-hybrid analysis of TaPP2C-a6/a7 and TaSnRK2s and the subcellular localization analysis of TaPP2C-a6. (**a**) The interactions of pGADT7-TaPP2C-a6 and pGBKT7-TaSnRK2s. (**b**) The interactions of pGADT7-TaPP2C-a7 and pGBKT7-TaSnRK2s. Positive transformants were cultured on selective medium DDO, TDO and QDO. Interactions between SV40-T and p53 or Lamin-C were set as positive or negative control, respectively. Three independent experiments were performed three times with similar results. (**c**) The recombinant vector pBI121-TaPP2C-a6-1D-GFP and control vector pBI121-GFP were transformed into the tobacco leaf epidermal cells, separately, and the green fluorescence signals were observed using fluorescence microscopy. The experiments were repeated three times (with similar results), and representative images are presented. Scale bar represents 10 μm.


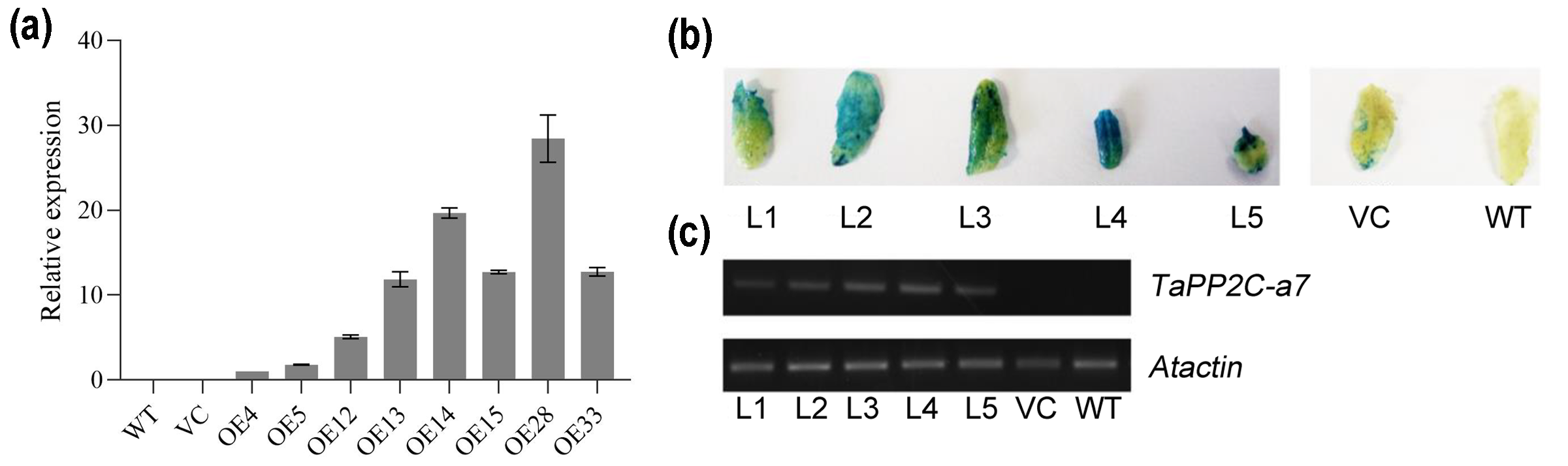


Figure S7. Quantification of the *TaPP2C-a6/a7* expression levels among the transgenic Arabidopsis lines. (**a**) The expression levels of *TaPP2C-a6* were analyzed by using the leaves tissues with the qRT-PCR assay. WT, wildtype, VC, vector control. OE, overexpression lines. *AtActin* was used as the internal reference gene. Data are presented as means ± S.E.M. for three biological replicates. (b) GUS staining results of *TaPP2C-a7* transgenic Arabidopsis lines (L1-L5), VC and WT. (c) RT-PCR analysis of *TaPP2C-a7* in leaves from *TaPP2C-a7* transgenic lines, VC and WT. *AtActin* was used as the internal reference gene. The GUS staining and RT-PCR experiments were repeated three times (with similar results), and representative images are presented.


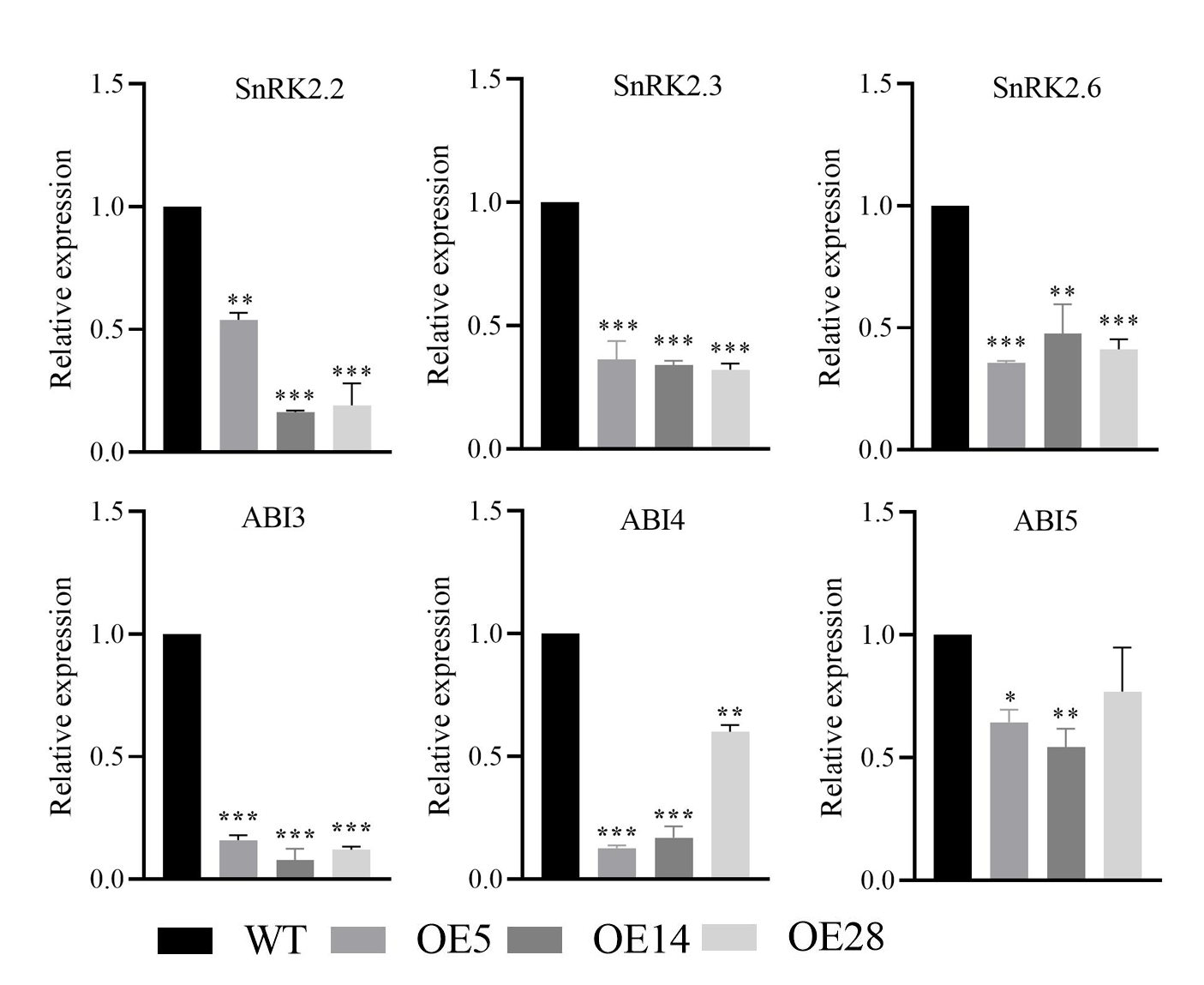


Figure S8. Expression analysis of the ABA-responsive genes in *TaPP2C-a6* overexpression and WT lines of Arabidopsis. The 7-day-old Arabidopsis seedling from 1/2 MS plates were used for the qPCR-based expression analysis. *AtActin1* was used as the internal reference gene. Data are presented as means ± S.E.M. of three biological replicates. The asterisks indicate significant differences in expression levels compared with that of WT. (Student’s *t*-test, **P*<0.05, ***P*<0.01, ****P*<0.001).


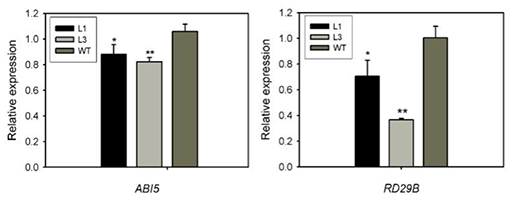


**Figure S9**. Expression analysis of the ABA-responsive genes in *TaPP2C-a7* overexpression and WT lines of Arabidopsis. The tissue, sampling stage and internal reference gene were the same as used for expression analysis of the *TaPP2C-a6* overexpression lines (mentioned in the caption of Figure S8). Data are presented as means ± S.E.M. of three biological replicates. The asterisks indicate significant differences in expression levels compared with that of WT. (Student’s *t* test, **P* <0.05, ***P*<0.01).


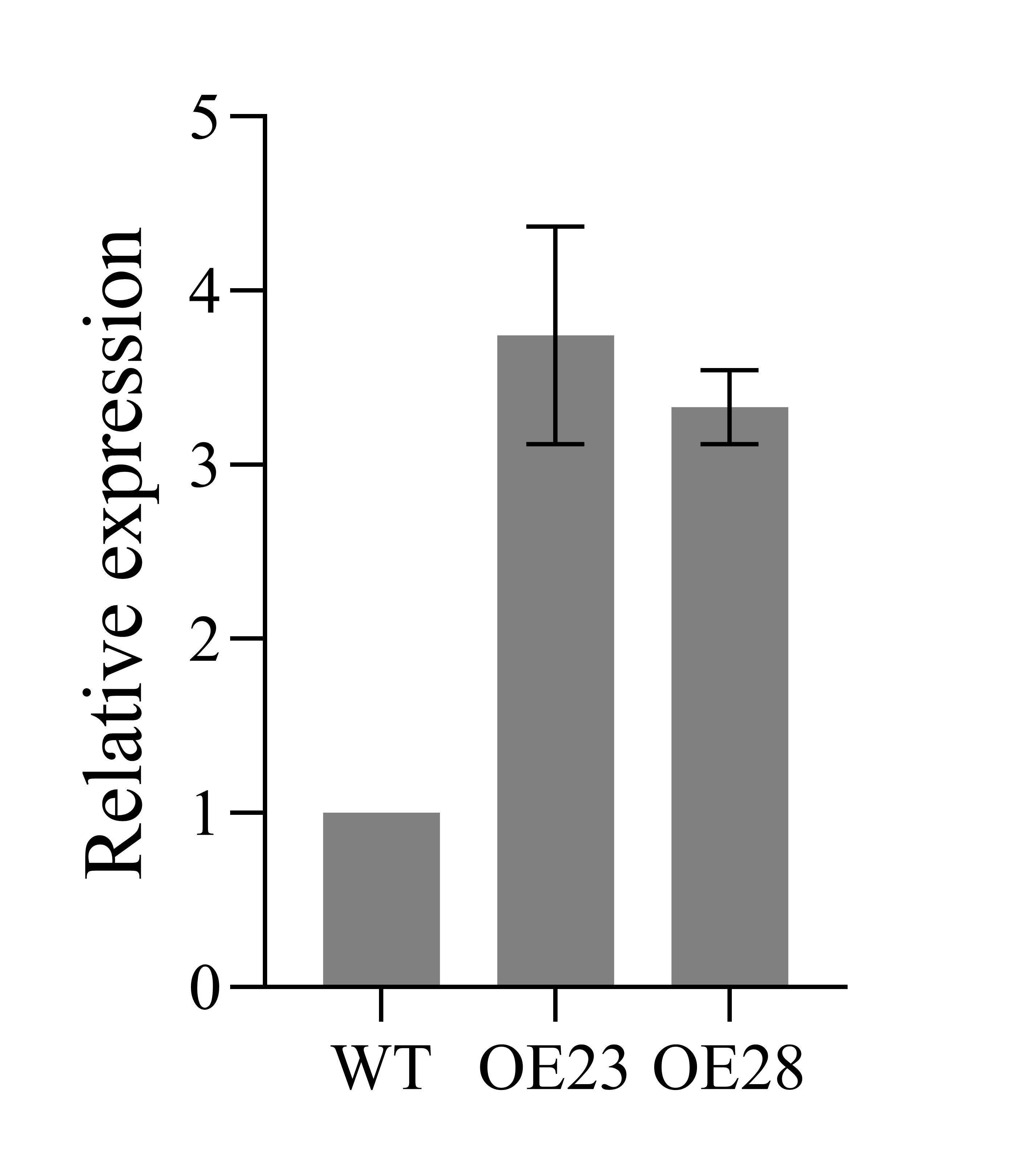


Figure S10. Quantification of the *TaPP2C-a6* expression levels among the transgenic wheat lines. The wheat seeds at 22 dpa from *TaPP2C-a6* transgenic lines and the non-transgenic wildtype (WT), respectively, were used for qPCR-based expression assay. This seed stage was chosen because it has a relatively high endogenous *TaPP2C-a6* expression level, therefore suitable for accurately identifying the transgenic wheat lines with high expression levels of transgenic *TaPP2C-a6*. *TaActin* was used as the internal reference gene. Data are presented as means ± S.E.M. of three biological replicates.


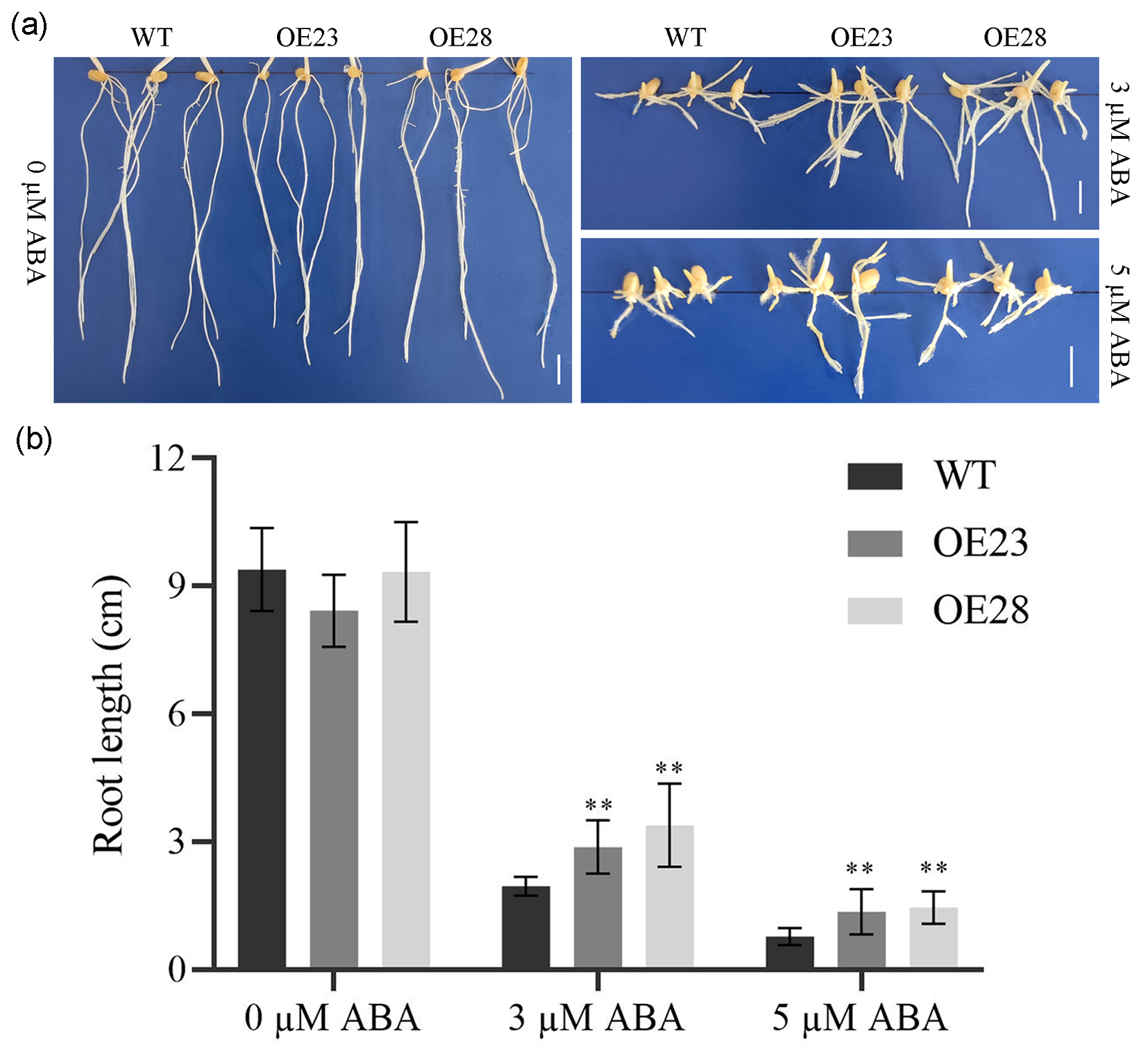


Figure S11. *TaPP2C-a6* overexpression in wheat reduces the ABA sensitivity during root growth. (a) Phenotypes of root growth of *TaPP2C-a6* OE and WT lines of wheat treated with 0, 3 and 5 μM ABA for seven days. Scale bar, 1 cm. (b) Comparisons between of root lengths of *TaPP2C-a6* OE and WT lines. Root lengths were measured after uniformed germinated seeds of *TaPP2C-a6* OE and WT lines were transferred to MS plates containing 0, 3 and 5 μM ABA for seven days. Data are presented as means ± S.E.M. of three biological replicates. At least nine seedlings were analyzed for each replicate. The asterisks indicate significant differences compared with WT. (Student’s *t* test, **P*<0.05 and ***P*<0.01).


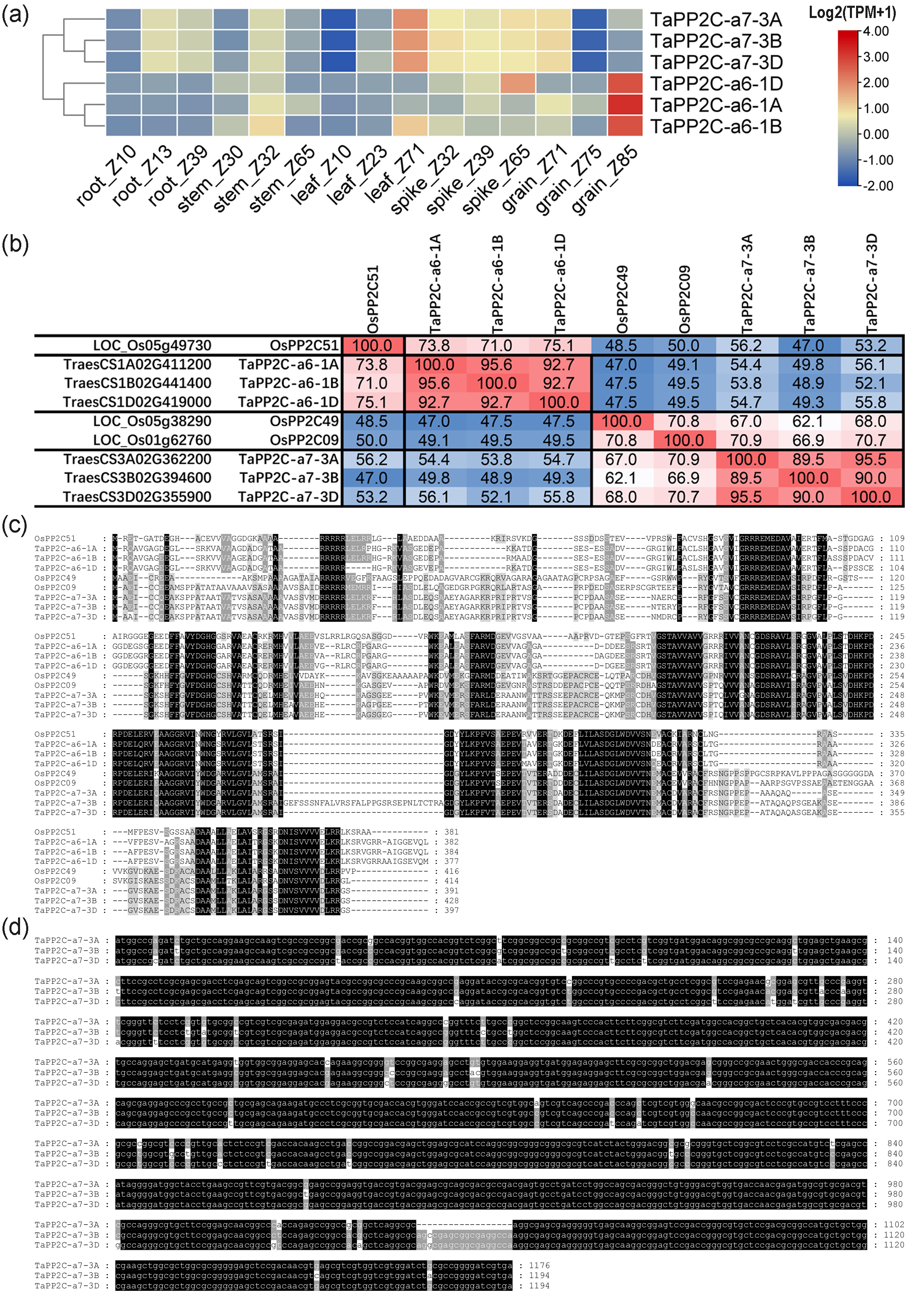


Figure S12. Tissue-specific expression and sequences identity analysis of *TaPP2C-a6* and *TaPP2C-a7*. (**a**) Expression profiles of *TaPP2Ca6* and *a7* in wheat various tissues (root, leaf, stem, spike and grain) (IWGSC, 2014). Red and blue cells denote relative higher or lower expression levels, respectively. (**b**) Amino acid sequence identity analysis of *TaPP2C-a6* and *a7* homologues. (**c**) Amino acid sequences alignment of *TaPP2C-a6* and *a7* homologues. The invariant and conserved amino acids are colored with black and grey shadow, respectively. (**d**) The cds alignment of *TaPP2C-a7* homologues. The invariant and conserved nucleotides are colored with black and grey shadow, respectively.

Table S1. The genes encoding the core components of ABA signaling in wheat. The nomenclatures of the wheat genes encoding ABA signaling core components from different studies were unified (Mega *et al*., 2019; Lei *et al*., 2021; Mao *et al*., 2022; Yu *et al*., 2019). (provided in a separate EXCEL file)

Table S2. The high-confidence geneIDs within the linked region of PHS QTL *QPhs.wsu-1A.2*. The expression levels of the genes were retrieved from published RNA-seq dataset collected at the WheatOmics database (Ramírez-González *et al*., 2018; Wei *et al*., 2019; Ma *et al*., 2021). (provided in a separate EXCEL file)

**Table S3**. The high-confidence geneIDs within the linked region of PHS QTL *QPhs.wsu-1B.2*. The expression levels of the genes were retrieved from published RNA-seq dataset collected at the WheatOmics database (Ramírez-González *et al*., 2018; Wei *et al*., 2019; Ma *et al*., 2021). (provided in a separate EXCEL file)

Table S4. The differentially expressed genes (DEGs) identified in wheat seed and embryo samples that are located within the linked region of PHS QTL *QPhs.wsu-1A.2*. The expression levels of the genes were retrieved from published RNA-seq dataset collected at the WheatOmics database (Ramírez-González *et al*., 2018; Wei *et al*., 2019; Ma *et al*., 2021). Columns “embryo_EXPR” and “embryo_DEG” indicate the expressed genes and differentially expressed genes (14 dpa versus 25 dpa, |log_2_(FoldChange)|>1 and q value<0.05), respectively. (provided in a separate EXCEL file)

Table S5. The differentially expressed genes (DEGs) identified in wheat seed and embryo samples that are located within the linked region of PHS QTL *QPhs.wsu-1B.2*. The expression levels of the genes were retrieved from published RNA-seq dataset collected at the WheatOmics database (Ramírez-González *et al*., 2018; Wei *et al*., 2019; Ma *et al*., 2021). Columns “embryo_EXPR” and “embryo_DEG” indicate the expressed genes and differentially expressed genes (14 dpa versus 25 dpa, |log_2_(FoldChange)|>1 and q value<0.05), respectively. (provided in a separate EXCEL file)

**Table S6.** Information of the 240 wheat accessions used in the *TaPP2C-a6* haplotype analysis. (provided in a separate EXCEL file)

Table S7. Primers used in the present study. (provided in a separate EXCEL file)

**Reference:**

Lei, P., Wei, X., Gao, R., Huo, F., Nie, X., Tong, W., and Song, W. (2021). Genome-wide identification of PYL gene family in wheat: Evolution, expression and 3D structure analysis. *Genomics*, **113**, 854-866.

Ma, S., Wang, M., Wu, J., Guo, W., Chen, Y., Li, G., Wang, Y. *et al*. (2021) WheatOmics: a platform combining multiple omics data to accelerate functional genomics studies in wheat. *Mol. Plant*, **14**, 1965-1968.

Mao, H., Jian, C., Cheng, X., Chen, B., Mei, F., Li, F., Zhang, Y. *et al*. (2022) The wheat ABA receptor gene TaPYL1-1B contributes to drought tolerance and grain yield by increasing water-use efficiency. *Plant Biotechnol. J*. **20**, 846-861.

Mega, R., Abe, F., Kim, J.S., Tsuboi, Y., Tanaka, K., Kobayashi, H., Sakata, Y. *et al*. (2019) Tuning water-use efficiency and drought tolerance in wheat using abscisic acid receptors. *Nat. Plants*, **5**, 153-159.

Ramírez-González, R.H., Borrill, P., Lang, D., Harrington, S.A., Brinton, J., Venturini, L., Davey, M. *et al*. (2018) The transcriptional landscape of polyploid wheat. *Science*, **361**, eaar6089.

The International Wheat Genome Sequencing Consortium. (2014) A chromosome-based draft sequence of the hexaploid bread wheat (*Triticum aestivum*) genome. *Science*, 345, 1251788.

Wei, J., Cao, H., Liu, J., Fang, Y., Lin, C., Sun, R., Li, W. *et al*. (2019) Insights into transcriptional characteristics and homoeolog expression bias of embryo and de-embryonated kernels in developing grain through RNA-Seq and Iso-Seq. *Funct. Integr. Genom.* **19**, 919-932.

Yu, X.F., Han, J.P., Wang, E.F., Xiao, J., Hu, R., Yang, G.X. and He, G.Y. (2019) Genome-wide identification and homoeologous expression analysis of *PP2C* genes in wheat (*Triticum aestivum* L.). *Front. Genet*. **10**, 561.
